# Unveiling a unique microglial phenotype promoting oxidation in the iBRB: insights from single-cell transcriptomics in the NPDR rat model

**DOI:** 10.1186/s13578-026-01613-z

**Published:** 2026-06-19

**Authors:** Yifei Geng, Yunqi Zhang, Xiaoyu Xu, Yixin Wang, Yun Luo, Xiaobo Sun

**Affiliations:** 1 Institute of Medicinal Plant Development, Chinese Academy of Medical Sciences & Peking Union Medical College, No. 151, Malianwa North Road, Haidian District, Beijing, 100193 PR China; 2 Beijing Key Laboratory of Neuroinnovative Drug Research and Development of Traditional Chinese Medicine (Natural Medicine), Institute of Medicinal Plant Development, Chinese Academy of Medical Sciences & Peking Union Medical College, Beijing, China; 3 Key Laboratory of Bioactive Substances and Resources Utilization of Chinese Herbal Medicine, Ministry of Education, Institute of Medicinal Plant Development, Chinese Academy of Medical Sciences & Peking Union Medical College, Beijing, China; 4 Diabetes Research Center, Chinese Academy of Medical Sciences & Peking Union Medical College, Beijing, China

**Keywords:** Single-cell RNA sequencing, Non-proliferative diabetic retinopathy, Inner blood-retina barrier, Microglia, Oxidation

## Abstract

**Background:**

Early diagnosis and targeted treatment of inner blood-retinal barrier (iBRB) impairment in non-proliferative diabetic retinopathy (NPDR) present significant challenges. This study investigates the cellular heterogeneity and early lesions in the iBRB microenvironment.

**Methods:**

We created a single-cell transcriptional atlas of NPDR using retinas from Zucker Diabetic Fatty rats, focusing on the expression of cells within the iBRB microenvironment, particularly microglia. We performed cell-cell gene interaction analyses to investigate intercellular communications among different cell types in the iBRB. Additionally, we conducted differentiation potential and trajectory analysis, transcription factor regulatory network characterization, and enrichment analysis of microglia. We also employed transmission electron microscopy, immunofluorescence, and histological analysis to validate the hypothesis.

**Results:**

Based on retinal samples from NPDR rats which had retinal edema and kidney damage, and normal rats, we selected 36,821 cells for subsequent analysis using single-cell sequencing. We identified 669 cells in iBRB and microglia had the highest connectivity value by enrichment analysis. 980 differentially expressed genes related to microglia divide it into 4 subtypes. Among them, *Spp1* highly expressed microglia subtype 2 had the highest differentiation potential and was significantly upregulated after NPDR lesions. By integrating histological and biochemical analysis results, the roles of the subtype in the differentiation of microglia in the iBRB microenvironment, such as oxidative stress and apoptosis, were well validated.

**Conclusion:**

This study identified a novel microglia type in NPDR rats, offering valuable insight into targeted therapeutic strategies for NPDR patients and enhancing our understanding of microglial regulation within the iBRB.

**Supplementary Information:**

The online version contains supplementary material available at https://doi.org/10.1186/s13578-026-01613-z.

## Introduction

Diabetic retinopathy (DR) stands as a prevalent microvascular complication of diabetes mellitus and serves as the primary cause of adult blindness [1]. Non-proliferative diabetic retinopathy (NPDR) typically manifests in the early stages of this condition. However, as it progresses to proliferative diabetic retinopathy (PDR) marked by angiogenesis and fibrosis, irreversible and substantial damage to the retina occurs, eventually leading to permanent blindness [2]. Consequently, the identification and management of NPDR constitute a crucial public health objective within the scope of DR therapy [3]. NPDR is notably defined by retinal vascular dilation, tortuosity, capillary occlusion, and heightened permeability of retinal blood vessels. The intricate mechanisms underpinning these pathological attributes involve the disruption of the inner blood-retinal barrier (iBRB) [4], which is considered as a hallmark of various degenerative retinal diseases. The iBRB comprises the retinal vascular unit consisting of vascular endothelial cells and their closely associated pericytes and Müller cells [5]. Collectively, these components work to restrict the passage of solutes, fluids, and toxic substances, thereby upholding the regulation of the environment in the diverse functional areas [6].

Recently, literature has indicated that pericytes can release soluble factors that stimulate microglia proliferation [7]. Furthermore, microglia activation can breach the basement membrane of iBRB, engulfing endothelial cells and subsequently compromising the integrity of iBRB [8]. Modulating the over-activation of microglia via drug intervention can facilitate a shift in its anti-inflammatory properties, thereby mitigating retinal inflammation and reducing the expression of tight junction proteins like occludin, claudin-5, and Zona Occludens 1, ultimately restoring iBRB integrity [9]. Inhibiting the PI3K/AKT/STAT3/NF-kB signaling pathway also deactivates microglia, consequently reducing iBRB leakage and enhancing pericyte coverage [10]. Nevertheless, the precise manner in which microglia influence the integrity of the blood-retinal barrier and the onset of inflammation in NPDR remains inadequately understood.

Elucidating the genetic profiles of diverse microglial types, pericytes, endothelial cells, and Müller cells may shed light on the heterogeneity of microglia and the blood-retinal barrier. In recent years, advances in single-cell RNA sequencing (scRNA-seq) technology have facilitated the examination of spatial distribution and transcriptional diversity of molecular function within each cell population [11]. In a previous investigation, researchers identified a particular pericyte type strongly linked to vascular dysfunction through single-cell transcriptome sequencing and demonstrated that silencing Col1a1 in this pericyte type balanced neovascularization across all regions of the retina [12]. Another study identified a subset of GPNMB^+^ microglia with profibrotic properties in vitreoretinal fibrovascular membrane disease in PDR and investigated the differentiation of this microglia from retinal-resident microglia through Pseudotime analysis [13]. Furthermore, scRNA-seq revealed the beneficial overexpression of RalA Binding Protein 1 (RLBP1) in Müller cells for ameliorating neurovascular degeneration in DR [14]. While these studies offer insights into the pathogenesis of PDR, there is a notable dearth of relevant investigations pertaining to NPDR.

Considering that DR represents a major cause of blindness in adults with type 2 diabetes (T2D) [15], this study employed obese Zucker diabetic fat (ZDF) rats exhibiting pathological and metabolic alterations akin to those observed in human DR-as a model of DR associated with type 2 diabetes mellitus [16]. ScRNA-seq was utilized to delineate the molecular mechanisms and signaling pathways of ZDF rat microglia on the iBRB in NPDR. This approach imparts novel perspectives for the early prevention, diagnosis, and treatment of DR.

## Methods and materials

### Rats NPDR induction and sample preparation

Eight-week-old healthy male ZDF rats (*n* = 6) were obtained from Beijing Vital River Laboratory Animal Technology Co., Ltd. (Beijing, China). All of the ZDF rats were adapted to ventilated cages (temperature: 20–25 °C, relative humidity: 30%-50%) under a 12-h light/dark cycle and were given free access to food and water. Animal procedures were approved by the Institutional Animal Care and Use Committee of the Chinese Academy of Medical Sciences & Peking Union Medical College (SYXK 2023-0008). The experimental group ZDF(fa/fa) rats (*n* = 3) were fed a high-sugar and high-fat diet for 8 weeks and were fasted but watered after 12 h, to establish the diabetic rat model. In contrast, the control group ZDF(fa/+) rats (*n* = 3) was fed with normal diet. The composition of the high-fat diet was 26.85% protein, 16.71% fat, and 56.44% carbohydrate. If the tail vein blood glucose was higher than 11.9mmol/l, the type 2 diabetes model was successful. At 16 weeks of age, the animals were anesthetized with ketamine (80 mg/kg), and the eyeballs were removed and frozen at -80 ℃ for histopathological examination.

### Single-cell RNA library construction and raw data collection of retinal cells

We performed the single-cell transcriptome sequencing (10×Genomics) on six mouse retinal tissues. The corneoscleral margin was cut from the removed eyeball, the anterior segment and vitreous were removed and subsequently, the intact retina was slowly separated and placed in a protective solution. Briefly, the globes were removed and dissected in DMEM (Thermo Fisher Scientific, Waltham, MA) on ice. Retinal samples were followed by cell dissociation which was digested in Collagenase A (6.25 mg mL^− 1^), Dispase II (6.25 mg mL^− 1^), and DNase (62.5 µg mL^− 1^; Roche) for 15 min at 37℃. Then it was with the addition of 0.25% trypsin-EDTA (Gibco) for 5 min at 37 °C to fully dissociate cells and to prepare the single-cell suspensions.

Single-cell suspensions (2 × 105 cells/mL) with PBS (HyClone) were loaded onto a microwell chip using the Singleron Matrix^®^ Single Cell Processing System. Barcoding Beads are subsequently collected from the microwell chip, followed by reverse transcription of the mRNA captured by the Barcoding Beads and to obtain cDNA, and PCR amplification. The amplified cDNA is then fragmented and ligated with sequencing adapters. Individual libraries were diluted to 4 nM, pooled, and sequenced on Illumina novaseq 6000 with 150 bp paired-end reads. Raw reads were processed to generate gene expression profiles using Cell Ranger v7.0.0. Reads from the 10X library were mapped to mm10-2020-A. Reads with the same cell barcode, unique molecular identifiers (UMIs), and genes were grouped together to calculate the number of UMIs per gene per cell. The UMI count tables of each cellular barcode were used for further analysis.

### Data quality control and graph-based clustering

We used Scanpy v1.8.2 to conduct quality control, dimensionality reduction, and clustering under Python 3.7 on the mouse single-cell transcriptome data. We filtered the expression matrix by the following criteria for each sample dataset: (1) cells with lower than 200 gene count with top 2% gene count were filtered out; (2) cells with top 2% UMI count were excluded; (3) cells with mitochondrial content 5% were excluded; (4) genes expressed in less than 5 cells were excluded. These criteria resulted in 21,685 cells in ZDF rats and 15,136 cells in ZDF(fa/+) rats for the downstream analysis. Batch effect correction was performed using Harmony (v1.0) with a resolution parameter of 2.0. Then Principal component analysis, Uniform Manifold Approximation and Projection (UMAP) dimensionality reduction, and cell clustering were performed after the raw count matrix was normalized by total counts per cell and logarithmically transformed into a normalized data matrix. The top 2000 variable genes were selected by setting flavor = ‘seurat’. Principle Component Analysis (PCA) was performed on the scaled variable gene matrix, and the top 20 principle components were used for clustering and dimensional reduction. Cells were separated into 24 clusters by using the Louvain algorithm and setting the resolution parameter at 1.2.

### Cell type annotation and subtyping of major cell types

The cell type identification of each cluster was determined according to the expression of canonical markers from the reference database SynEcoSysTM (Singleron Biotechnology). SynEcoSysTM contains collections of canonical cell type markers for single-cell seq data, from CellMakerDB, PanglaoDB, and recently published literatures. To obtain a high-resolution map of Main / Muller Cells / Amacrine Cells / Bipolar Cells / Microglial Cells / Retinal Cone Cells / Retinal Rod Cells, cells from the specific cluster were extracted and reclustered for more detailed analysis following the same procedures described above and by setting the clustering resolution as 1.2/1/0.4/0.1/0.3/0.5/0.4.

### Differentially expressed genes (DEGs) identification and pathway enrichment analysis

Each cluster was further designated by the histology distribution and the DEGs identified using the scanpy.tl.rank_genes_groups() function based on Wilcoxon rank sum test with default parameters, and selected the genes expressed in more than 10% of the cells in either of the compared groups of cells (adjusted p value < 0.05 and average log2(Fold Change) value > 0.25). To investigate the potential functions of endothelial cells, microglia, pericytes, and Müller cells, Gene Ontology (GO) and Kyoto Encyclopedia of Genes and Genomes (KEGG) analysis were used with the “clusterProfiler” R package v 4.0.0 (ref). Pathways with p_adj value less than 0.05 were considered as significantly enriched. Selected significant pathways were plotted as bar plots. GSEA was performed in 8 clusters. For GSVA pathway enrichment analysis, the average gene expression of each cell type was used as input data. GO gene sets including molecular function (MF), biological process (BP), and cellular component (CC) categories were used as reference.

### Cell-cell gene interaction analysis

To visualize the intercellular communications of different types of cells in iBRB, we conducted cell-cell interaction between each type of cell cluster based on known ligand-receptor pairs by Cellphone DB [17] (v4.0.0) (ref) version. In detail, the permutation number for calculating the null distribution of average ligand-receptor pair expression in randomized cell identities was set to 1000. Individual ligand or receptor expression was thresholded by a cutoff based on the average log gene expression distribution for all genes across each cell type. Definitively, predicted interaction pairs with p-value < 0.05 and of average log expression > 0.1 were considered significant and visualized by heatmap_plot and dot_plot in CellphoneDB.

### Cell differentiation potential evaluation and pseudotime trajectory analysis

To understand the differentiation and development of each cell subset of microglia in the pathological process of NPDR, we performed differentiation ability and developmental trajectory prediction. CytoTRACE v0.3.3 (ref) (a computational method that predicts the differentiation state of cells from single-cell RNA-sequencing data using gene counts and expression [18]) was used to predict the differentiation potential of cell subpopulations. Monocle2 v2.22.0 (ref), Monocle3 v1.0.0, and RNA velocity was used to reconstruct cell differentiation trajectory. For constructing the trajectory, the top 2000 highly variable genes were selected by Seurat(v4.1.0) FindVairableFeatures(), and dimension-reduction was performed by DDRTree(). The trajectory was visualized by the plot_cell_trajectory() function in Monocle2. DEGs were used to sort cells in order of spatial-temporal differentiation. We used UMAP to perform graph_test() and dimension-reduction and recognition trajectory by the learn_graph() function. Finally, the trajectory was visualized by the plot_cells() function in Monocle3. For RNA velocity, the BAM file containing every subtype of Microglias and reference genome GRCm38 (mm10) were used in the analysis with velocyto(v0.17.17) and scVelo (v0.2.4)in Python with default parameters [19]. The result was projected to the UMAP plot from the Seurat clustering analysis for visualization consistency.

### Transcription factor regulatory network analysis in single-cell data

To further explore the differences between the simultaneous gene regulatory network and cell state in the microglia subsets, we applied scenic (v0.11.0) [20] using scRNA expression matrix and transcription factors in AnimalTFDB to mine the transcription factors (TFs). First, GRNBoost2 predicted a regulatory network based on the co-expression of regulators and targets. CisTarget was then applied to exclude indirect targets and to search transcription factor binding motifs. After that, AUCell was used for regulon activity quantification for every cell. Cluster-specific TF regulons were identified according to Regulon Specificity Scores (RSS) and the activity of these TF regulons were visualized in heatmaps.

### UCell gene set scoring

To explore the unique functions of microglia subsets, we used UCell to score the gene sets, and the results were presented as UMAP and violin plots. Gene set scoring was performed using the R package UCell v2.2.0 [21]. UCell scores are based on the Mann-Whitney U statistic by ranking query genes’ in order of their expression levels in individual cells. Because UCell is a rank-based scoring method, it is suitable to be used in large datasets containing multiple samples and batches.

### Fundus fluorescein angiography (FFA)

After being anesthetized with 1% pentobarbital sodium and 10% sulfanilamide injection, rats received epinephrine dilute eye drops to dilate their pupils and carbomer eye drops to protect them. Then, 10% fluorescein was injected into the peritoneum, and 2 min later, a digital fundus camera (Retina Imaging System, OptoProbe Research Ltd., Burnaby, Canada) was used to identify FFA. Based on our latest research, we collected fluorescent images of the retina and used the AngioTool software (1.8.0 version) to calculate the vessel density [22].

### Glucose metabolism detection and histological analysis

To identify whether glucose metabolism disorders in diabetes ZDF rats, we determined the levels of kidney urea (UREA) (Abcam, ab83362, USA) and crea (CRE) (Abcam, ab65340, USA), lactate dehydrogenase (LDH) (Mlbio, Shanghai, China) and creatine kinase isoenzymes-MB (CK-MB) (Mlbio, Shanghai, China), liver alanine aminotransferase (ALT) (Abcam, ab234579, USA) and AST (Abcam, ab263883, USA) by enzyme-linked immunosorbent assay (ELISA). Plasma levels of glucose, superoxide dismutase (SOD) (Mlbio, Shanghai, China), adenosine triphosphate (ATP) (Abcam, ab287863, USA), malonaldehyde (MDA) (Mlbio, Shanghai, China), Acetyl-CoA (CoA) (Mlbio, Shanghai, China) and were also measured by ELISA, followed by the provided assay protocol. Hematoxylin-eosin staining (HE stain) was used to check the pathological changes of the retina. Rats were anesthetized by injecting 1% pentobarbital sodium and 10% sumianxin, a part of the rats received tropicamide phenylephrine eye drops to enlarge their pupils and carbomer eye drops to shield them. Immediately after an intraperitoneal injection of 10% fluorescein sodium (Solarbio), FFA was identified using a digital fundus camera at the 2-minute interval (Retinal Imaging System, OptoProbe Research Ltd., Burnaby, Canada). Another part of the rats was harvested from the retinas under a stereomicroscope. The dehydrated retinas were fixed with 4% paraformaldehyde and embedded in paraffin Sect.  (5 μm) which were stained using HE. Retinal histopathology pictures were obtained by a digital slide image scanning method (Aperio CS2, Leica, Germany).

### Transmission electron microscopy

After sacrificing the subjects, eyeballs were quickly removed and fixed in 2.5% glutaraldehyde (Solarbio) for 2–4 h. Then the retinachoroid-sclera complex was dissected, cut into small 1 mm3 clumps, post-fixed with 1% osmium tetroxide in a 0.1 M phosphate buffer (PB, pH 7.4) at room temperature for 2 h, dehydrated in a graded ethyl alcohol series, and embedded in SPI-Pon 812 epoxy resin (SPI, Cat#90529-77-4, PA, USA) overnight at 37 ◦C. The embedded tissues were polymerized for more than 48 h at 60 ◦C. Then 60–80 nm ultra-thin sections were cut using a Leica UC7 ultramicrotome, stained with 2% uranium acetate saturated alcohol solution for 8 min and 2.6% lead citrate for 8 min, and observed under an HT7700 transmission electron microscope (HITACHI, Japan).

### Immunohistochemistry and immunofluorescence

After euthanasia, eye globes were fixed for 30 min in 4% paraformaldehyde in PBS. Fixed eyes were then incubated overnight in 30% sucrose at 4 °C. Retinas were then frozen in optimal cutting temperature compound ( O.T.C.) and cryosectioned at 18 μm. For immunostaining, sections were washed two times for 10 min in PBS and then incubated in primary antibody in 0.5% Triton X-10 and PBS overnight. Slides were then washed in PBS and incubated in secondary antibodies for 1 h. Last, slides were washed again in PBS and coverslipped with Fluoromount-G (SouthernBiotech). All the andibodies were form Proteintech (Wuhan, China). The ratio of aggregates/monomers of mitochondrial membrane potential (MMP) in R28 cells was assessed by using the dye JC-1 (C2003S, Beyotime, China). Cells were incubated in accordance with the manufacturer’s instructions then measured by utilizing a fluorescence microscope (Leica, Wetzlar, Germany). Images were analyzed and merged by using ImageJ software.

Cell culture and testingBV2 cells (CL-0493, Procell, China) were cultured at a density of 1 × 10^5^/ml. siRNA was transfected using lipo3000 (L3000001, Thermo, America). After 24 h of stimulation with 1 µg/mL LPS (S1732, Beyotime, China), cell viability was detected using the CCK-8 kit (96996, Sigma, Germany), and cell fluorescence intensity was measured using the ROS kit (S0033M, Beyotime, China).

### Statistical analyses

Data were analyzed with GraphPad Prism 9.5.0 (GraphPad Software, San Diego, CA) and are presented as the mean ± SD. Statistical differences between the two groups were compared with an unpaired two-tailed Student t-test, and those between multiple groups were examined with ANOVA with a Bonferroni correction. *P* < 0.05 was considered statistically significant.

## Results

### NPDR models were established and Single-cell profiles of retinas were obtained from ZDF (fa/fa) rats and ZDF(fa/+) rats

**Fig. 1:**
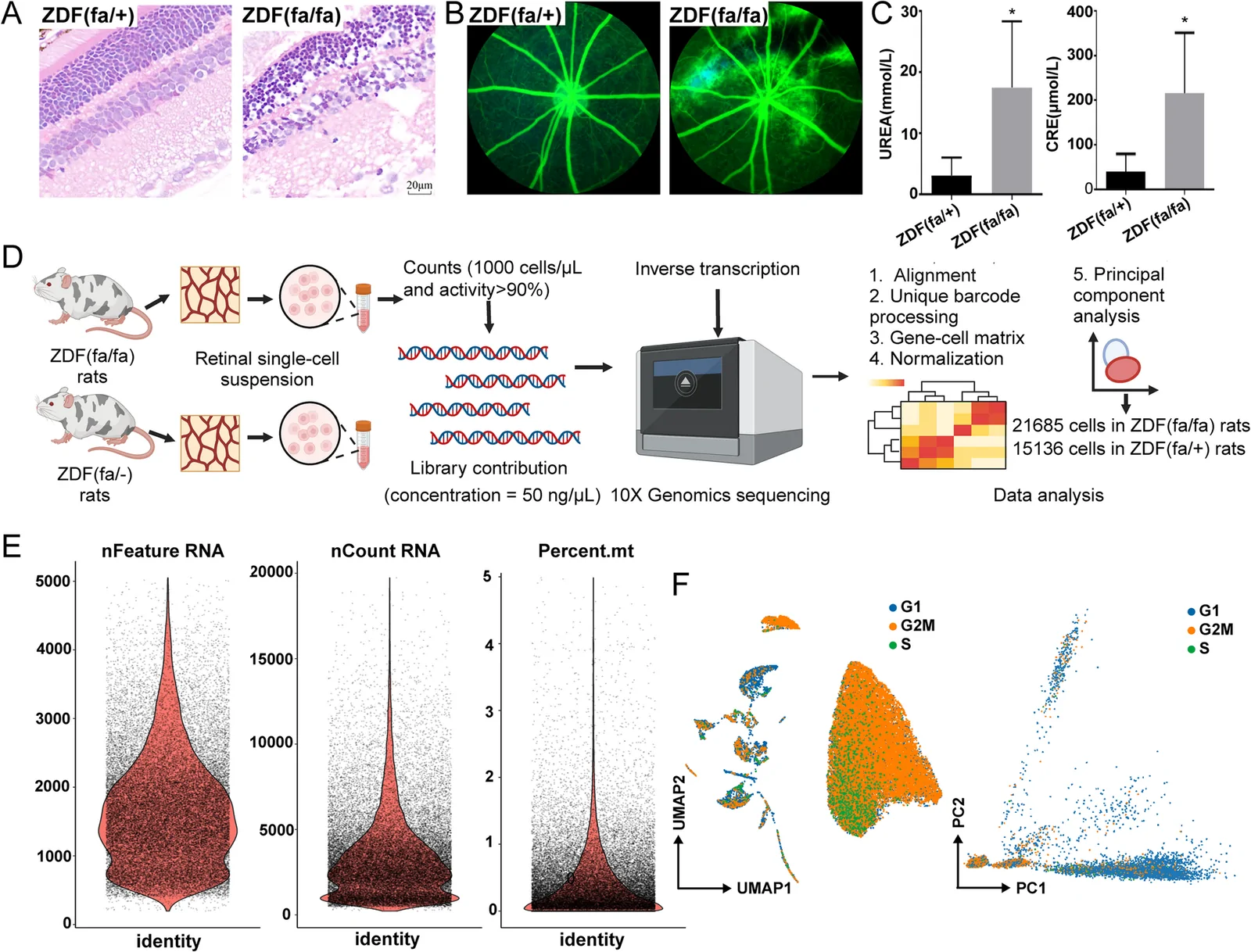
Single-cell profile of retinas obtained from ZDF(fa/+) and ZDF(fa/fa) rats under NPDR. **A** HE stained sections of retinas from ZDF(fa/+) and ZDF(fa/fa) rats (*n* = 5). **B** Fundus fluorescein angiography images of the retina in ZDF(fa/+) and ZDF(fa/fa) rats (*n* = 5). **C** Serum urea and serum creatinine(CRE) contents in ZDF(fa/+) and ZDF(fa/fa) rats (*n* = 5). **D** The flow of retinal isolation, single-cell transcriptome sequencing, and library building analysis. The ZDF(fa/fa) rats were fed a high-fat diet, and the ZDF(fa/+) rats were fed a normal diet. After 90 days, the retinas were collected and prepared into single-cell suspension with a concentration of 1000 cells /µL and a cell activity of more than 90%, and the concentration of RNA for library was 50ng/µL. 10X genomics sequencing platform was used for sequencing, and the data were processed and normalized (*n* = 3). **E** Violin plot showing gene number in each cell (nFeature), total count number of all genes in each cell (nCount), and percentage of mitochondrial gene number in total gene number in each cell (percent.mt). **F** UMAP and PCA maps of cell cycle distribution in G1, G2M and S phases. (*P < 0.05)

We tested the success of the NPDR model after 8 weeks by feeding ZDF(fa/ +) rats on a regular diet and ZDF(fa/fa) rats on a high-fat diet. Notably, the retinal morphological characteristics of ZDF(fa/fa) rats displayed significant differences compared to those of ZDF(fa/+) rats. In the former, the retinal cells were disorganized and exhibited edema, alongside increased vascular permeability and neovascularization (Figs. 1A-B). Furthermore, ZDF(fa/fa) rats demonstrated more severe liver and kidney damage, as well as markedly elevated myocardial enzyme activities compared to their ZDF(fa/+) counterparts (Fig. 1C, Supplementary Fig. 1A-B).To investigate the structural changes and cellular heterogeneity in the retina during NPDR, we prepared highly reactive single-cell suspensions containing 1,000 cells per microliter from ZDF(fa/fa) rats with the fa/fa genotype, subjected to a high-fat diet, and from ZDF rats with the fa/- genotype, maintained on a normal diet. Subsequent single-cell sequencing and data analysis were conducted (see Fig. 1A). A total of 21,685 cells were obtained from three ZDF (fa/fa) rats, while 15,136 cells were collected from three ZDF (fa/+) rats.

For data selection, we employed three indicators: the number of RNA molecules in each cell, the total count of all RNA within each cell, and the ratio of mitochondrial gene counts to total gene counts per cell. Most samples contained approximately 3,500 genes, with sequencing counts for all samples averaging below 100,000. Additionally, the percentage of mitochondrial genes (Percent.mt) across nearly all samples remained under 25% (Fig. 1E). Importantly, the influence of the cell cycle on dimensionality reduction was successfully mitigated in the analyses (Fig. 1F), indicating that the data underwent rigorous quality control and that the model was effectively established.

### Single-cell sequencing reveals the dominant role of microglia in iBRB injury

**Fig. 2:**
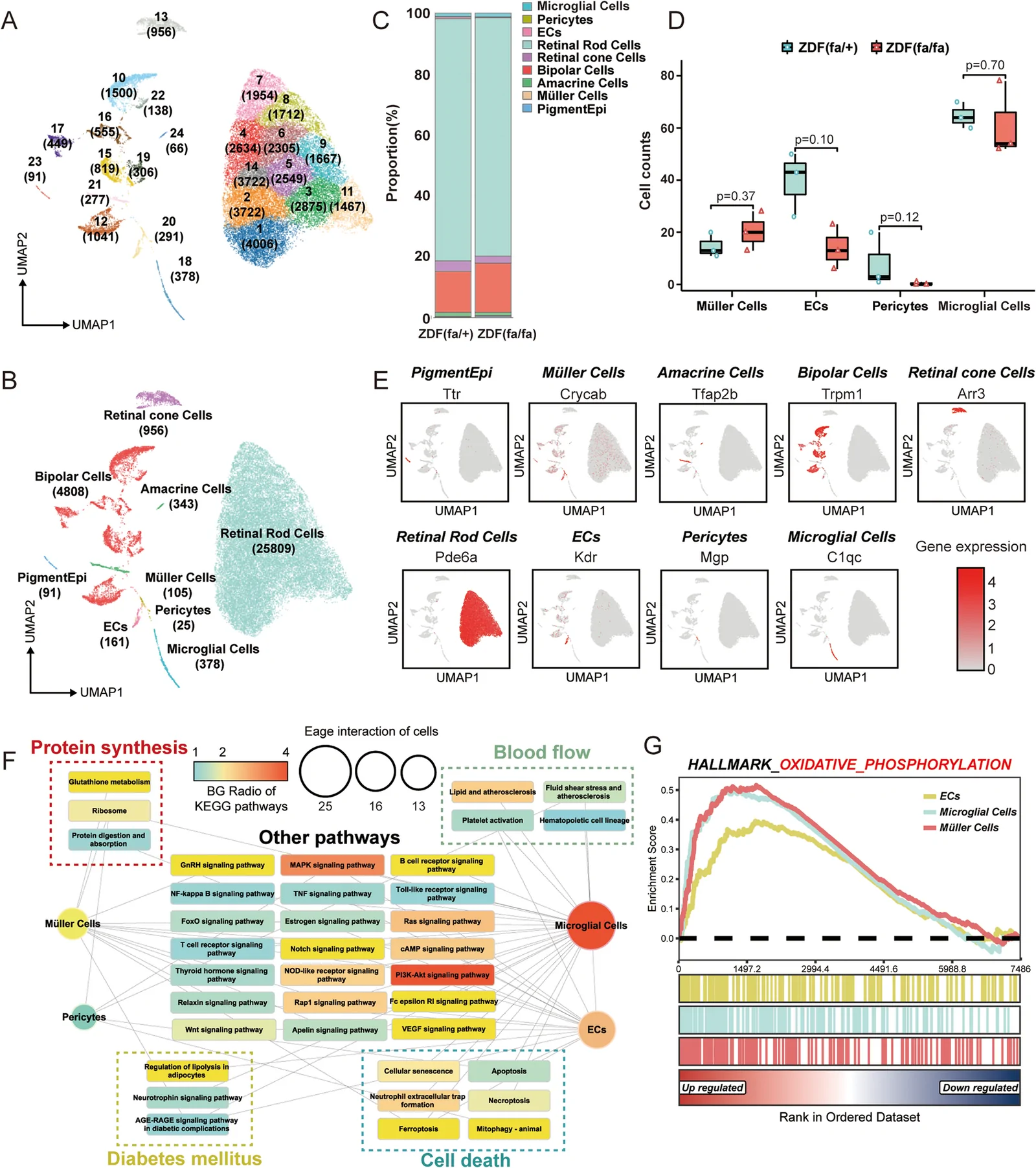
Determination of retinal cell clusters and cell populations in the iBRB microenvironment function. **A**-**B** UMAP representation of single-cell gene expression showing 23 cell clusters and 9 identified retinal cell types. Single cells were color-coded by cluster annotation. **C** The proportion of the 9 cell types in the retina of ZDF(fa/+) and ZDF(fa/fa) groups. **D** Box plot of the proportion of endothelial cells, microglia, pericytes, and Müller cells in the ZDF(fa/+) and ZDF(fa/fa) groups. **E** UMAP plot of the Lyon distribution of marker genes for each cell type, with darker colors showing higher expression. **F** KEGG enrichment analysis pathway map of differential genes of four kinds of cells in iBRB microenvironment, square represents pathways, circle represents cells, color from green to red, representing more enriched pathways or more enriched cells. **G ** GSEA plot of oxidative phosphorylation in endothelial cells, microglia, and Muller cells, with each colored band representing a cell population

Single-cell sequencing has illuminated the prominent function of microglia in the injury of the inner blood-retinal barrier (iBRB). Utilizing the most variable DEGs, a t-distributed Stochastic Neighbor Embedding (t-SNE) map comprising 24 clusters was generated (Fig. 2A). Further analysis identified 12 distinct cell populations within the retina of ZDF rats: amacrine cells (cluster 24), bipolar cells (clusters 10, 15, 16, 17, 19, and 22), cone cells (cluster 13), endothelial cells (part of cluster 20), Müller cells (part of cluster 20), microglia (cluster 18), pericytes (part of cluster 20), and rod cells (clusters 0 through 11 and 14) (Fig. 2B and Supplementary Fig. 2). The annotated genes of each cell cluster are presented in Fig. 2E.

In the state of NPDR, the population of Müller cells within the iBRB increased, while the numbers of pericytes, endothelial cells, and microglia decreased (Figs. 2C-D). We examined the DEGs between ZDF(fa/+) and ZDF(fa/fa) rats, revealing enriched pathways in the aforementioned four cell types. Intriguingly, within the microenvironment of the iBRB, pathways associated with protein synthesis, blood flow, diabetes, and cell death were significantly enriched. Notably, both the MAPK and PI3K-Akt signaling pathways were implicated in the processes affecting these four cell types. Among them, microglia exhibited the highest enrichment of DEGs, underscoring their indispensable role in the pathogenesis of the disease (Fig. 2F, Supplementary Table 1).

Moreover, Gene Set Enrichment Analysis (GSEA) indicated significant upregulation of oxidative phosphorylation pathways in Müller and endothelial cells following NPDR in rats (Fig. 2G). This surge in oxidative phosphorylation could lead to an imbalance in the formation and clearance of free radicals, culminating in oxidative stress (OS). Numerous studies have implicated OS as a crucial pathogenic factor in diabetic retinopathy (DR), resulting in increased expression of vascular endothelial growth factor and subsequent inflammation, which damages retinal microvessels [23, 24]. Disruption of the iBRB, characterized by enhanced vascular permeability and angiogenesis, has also been shown to be mitigated by reducing reactive oxygen species (ROS) release from microglia [25].

**Fig. 3:**
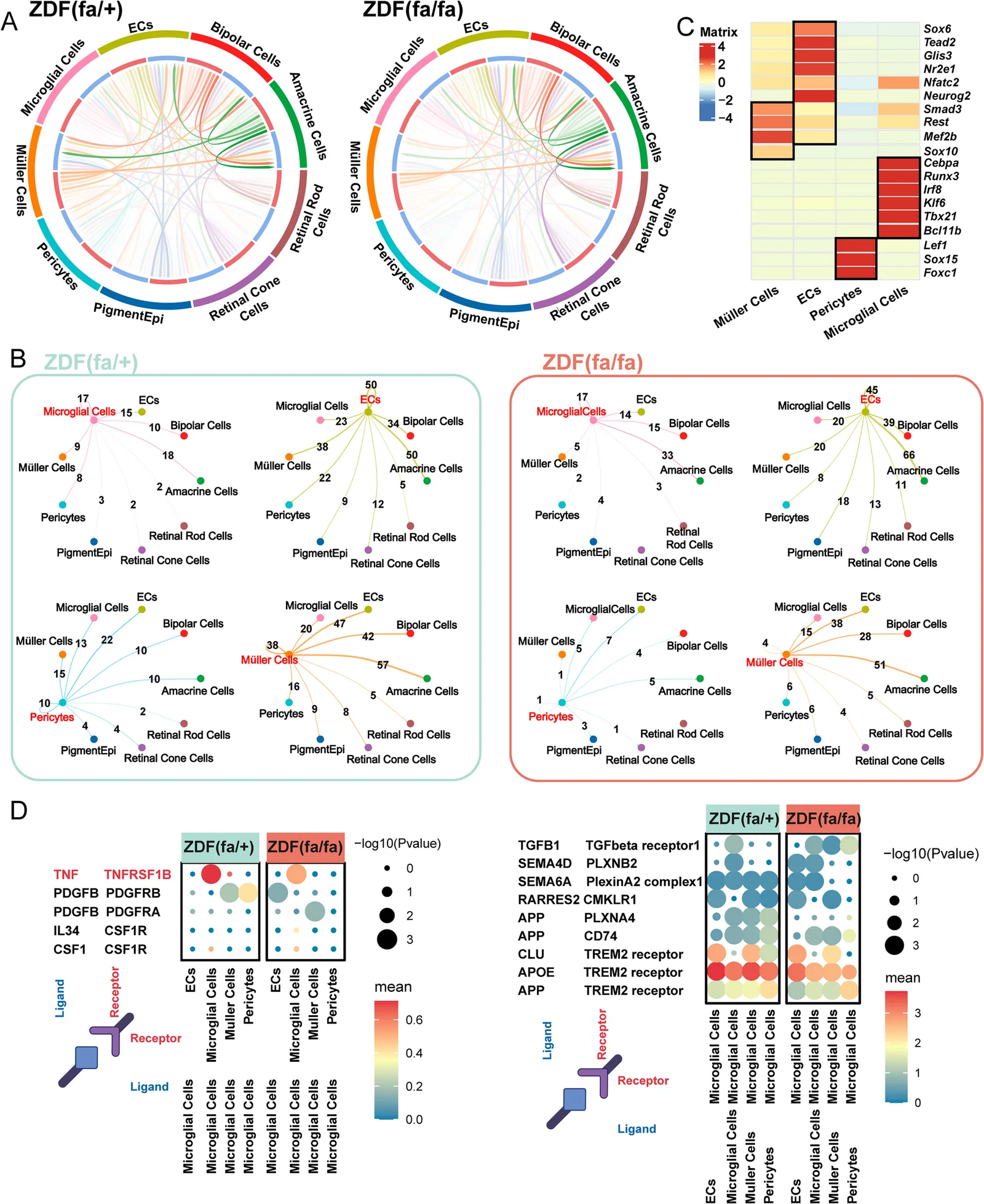
Cell-cell interactions and transcriptional regulation in the iBRB microenvironment. **A** Chord diagram of cell interactions showing the number of interaction pairs between cell types. The outer color block represents the cell type. The inner circle red represents the ligand and the blue generation Surface receptor; Line sharpness was positively correlated with the number of interaction pairs between the two cell types, with the greater the number the clearer. **B** The line color was consistent with the ligand cell type, and the line thickness was positively correlated with the number of interaction pairs when each of the four cell types was used as a ligand cell. **C** The AUC matrix clustering heatmap of top3-6 regulon in each cell in the four cell types. **D** Bubble plot of the predicted interactions between microglia as ligand and receptor cells and other three cell types

To further explore transcription factor enrichment and regulatory activity among various cell types in the iBRB microenvironment, we employed pyscenic analysis. In ZDF rats, the top four transcription factors identified in Müller cells were *Mef2b*, *Rest*, and Smad3; the top five for endothelial cells included *Neurog2*, *Tead2*, *Glis3*, *Nr2e1*, and *Sox6*; the predominant transcription factors for pericytes were *Lef1*, *Sox15*, and *Foxc1*; and for microglial cells, the most relevant factors were *Cebpa*, *Runx3*, *lrf8*, *Kif6*, *Tbx21*, and *Bd11b* (Fig. 3C). In the comparative analysis of transcription factor expression within these four cell types between control and model groups, the expression profiles in ZDF(fa/+) and ZDF(fa/fa) rats were divergent for each corresponding cell type (Supplementary Fig. 4). These changes in transcription factors suggest signaling crosstalk between microglia and the other three cell types. However, the specific expression of these transcription factors require experimental validation.

Next, we aimed to elucidate the interactions between microglia and other cellular types in the iBRB during the progression of NPDR. CellphoneDB analysis revealed a decrease in receptor-ligand interactions among microglia, endothelial cells, pericytes, and Müller cells during DR (Fig. 3A). However, the number of ligands in microglia remained unchanged within the iBRB microenvironment (Fig. 3B), suggesting that cell-to-cell interactions among microglia and the other three clusters did not diminish. Specifically, when microglia functioned as ligands, TNF-TNFRSF1B emerged as the prominent mediator in microglial communications, with diminished effects observed in ZDF(fa/+) rats compared to ZDF(fa/fa) rats. TNFRSF1B has been identified as a specific and sensitive biomarker in regulatory T cells of children with Type 1 Diabetes (T1D) [26]. Its genetic variations may predispose individuals to clinical neuropathy in T2D patients [27]. Notably, significant associations have been reported between DNA methylation of TNFRSF1B and glycosylation in gestational diabetes mellitus [28].

Furthermore, interactions involving PDGFB-PDGFRB also decreased in microglia-Müller cell communications. Microglia are critical players in vascular pathology associated with NPDR. When functioning as receptors, the TGFB1-TGFBR1 and SEMA4D-PlexinA2 complexes were enhanced in ZDF(fa/fa) rats. Previous insights indicate that TGFB1 is involved in the destruction of the blood-retinal barrier in diabetic conditions and promotes [29, 30]. At the same time, inhibiting SEMA4D alleviates vascular dysfunction and leakage [31]. Expression of activation-related gene clusters in inflammatory microglia and reactive microglia was reduced in ZDF(fa/fa) rats, as the interactions of APP-PLXNA4, APP-CD74, CLU-TREM2 receptor, APOE-TREM-receptor, and APP-TREM2 receptor were weakened (Fig. 3D). Thereby, we detected that microglia in the retina of ZDF(fa/fa) rats play critical roles in iBRB, participating in the proliferation and leakage of neovascularization after NPDR lesions, and genetic changes in microglia before and after diabetic retinopathy may be potential biomarkers.

### Identification of activated *Spp1*^**+**^ microglia as the origin of iBRB damage in NPDR

The activation of microglia plays a crucial role in the increase of vascular permeability and the breakdown of the iBRB [9]. Nonetheless, the transcriptomic and signaling heterogeneity of pericytes in the context of NPDR remains inadequately understood. We subsequently isolated microglia based on their marker genes and conducted detailed analyses to characterize the sub-populations of these cells. A dimensionality reduction approach utilizing UMAP was employed to illustrate the heterogeneity within the microglial population, which was delineated into four distinct sub-populations.

**Fig. 4:**
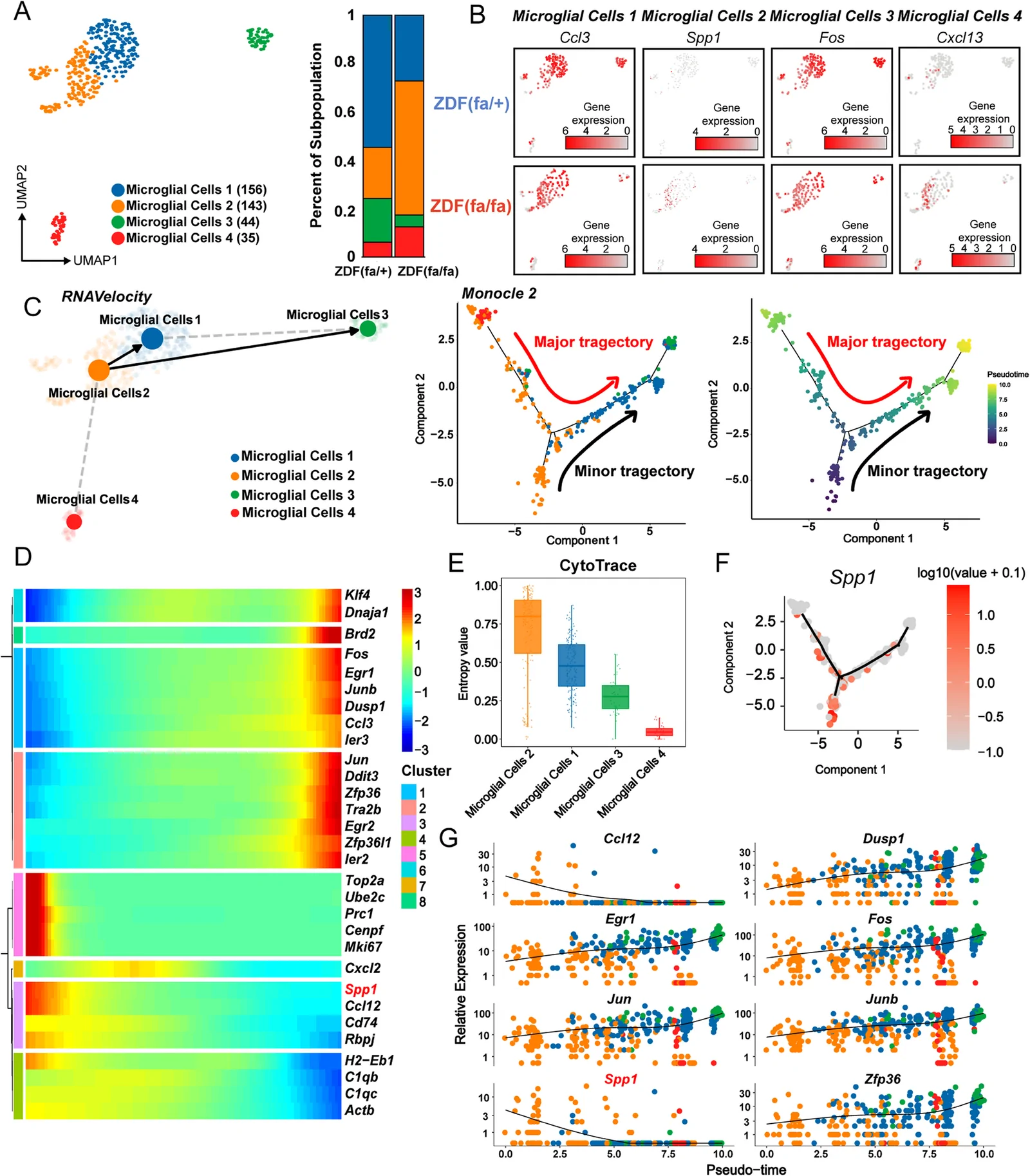
Microglia (2) is the source of microglia in the iBRB microenvironment in ZDF rats. **A** UMAP of the distribution of each microglial subpopulation and the proportion of cell populations in ZDF(fa/+) and ZDF(fa/fa) rats (**B**) UMAP plot of Top 1 marker genes expressed in ZDF(fa/+) and ZDF(fa/fa) rat retinal cells for four microglial subsets. **C** Display plot of the differentiation direction of each cell in the RNAVleocity analysis and Monocle 2, with arrows representing the direction of differentiation, the abscissa represents the order of pseudotime **D** A situation in which the expression of a gene changes as a function of pseudo time. The abscissa from left to right is the pseudotime from small to large, the ordinate is the expression of the gene, and different colors indicate different cell types. **E** Boxplots of entropy values /CytoTrace scores for four microglial subsets. The abscissa represents the different cell types, and the ordinate is the entropy value CytoTrace score of the corresponding cell type. Higher entropy /CytoTrace scores represent higher stemness and higher differentiation potential of cells. **F** The expression of the Spp1 gene was displayed in the monocle dimensionality reduction (DDTree) graph. **G** Changes in the expression of the top 8 genes (Reverse order of genes according to q value) as a function of pseudo time. The abscissa from left to right is the pseudo-time from small to large, the ordinate is the expression of the gene, and different colors indicate different cell types

To ascertain which microglial sub-population is the primary contributor to iBRB breakdown and is particularly susceptible to pathological angiogenesis and leakage in NPDR, we compared the proportions of various microglial sub-populations in wild-type (ZDF(fa/+)) and ZDF(fa/fa) rats. We observed a significant upregulation of the Microglia (2) marker gene *Spp1* and the Microglia (4) marker gene *Cxcl13*, alongside a downregulation of the Microglia (1) marker *Ccl3* and the Microglia (3) marker *Fos* in ZDF(fa/fa) rats compared to ZDF(fa/+) rats. (Fig. 4A-B). Meanwhile, our data indicate that the gene expression levels of Spp1, Ccl3, and Cxcl13 are the highest in microglial cells and are significantly higher than those in other cell types. Although the gene expression level of Fos is not the highest in microglial cells, it is still at a relatively high level (Supplementary Fig. 3B).

To investigate the ontogeny and cellular dynamics of microglia, we examined the distribution of each microglial subtype along a pseudotemporal trajectory. The transcriptional profiles from the single-cell transcriptomic datasets were mapped out along this pseudotemporal continuum. Both RNA velocity and Monocle 2 analyses indicated that the *Spp1*^+^ Microglia (2) cells are positioned at the origin of this trajectory, gradually transitioning into either Microglia (1) or Microglia (3) sub-populations. Interestingly, Microglia (4) was also linked with the Microglia (2) cluster on the UMAP plot, illustrating a major trajectory that indicates shared fates dictated by two distinct differentiation pathways (Fig. 4C). CytoTrace analysis revealed notable differences in differentiation levels among each sub-population, with Microglia (2) demonstrating significantly greater differentiation potential than the other sub-populations (Fig. 4E). To explore transcriptional heterogeneity during differentiation, we utilized Monocle 2 to identify DEGs across sub-populations before and after disease progression along the pseudotemporal axis. The expression levels of genes such as *Klf4*, *Dnaja1*, *Brd2*, *Fos*, *Egr1*, *Junb*, *Dusp1*, *Ccl3*, *Ier3*, *Jun*, *Ddit3*, *Zfp36*, *Tra2b*, *Egr2*, *Zfp36l1*, and *Ier2* increased steadily over time. Consistent with earlier findings, the significant expression of the Microglia (2) marker gene *Spp1* was observed at the initial stages of differentiation, followed by a gradual decline. Similar patterns were noted for *Top2a*, *Ube2c*, *Prc1*, *Cenpf*, *Mli67*, *Cxcl2*, *Ccl12*, *Cd74*, *Ebpj*, *H2-Eb1*, *C1qb*, *C1qc*, and *Actb* (Fig. 4D). Monocle 2 highlighted the metastatic trajectory of *Spp1* throughout the pseudotemporal progression (Fig. 4F). We performed immunofluorescent labeling of these four microglial subtypes in the retinas of NPDR rats. The staining results indicated that *Spp1*^+^ microglia did not co - localize with the other three microglial subtypes and the expression level of *Spp1* in microglial cells is significantly higher than that in other types of cells. (Supplementary Fig. 3A-B), suggesting that *Spp1*^+^ microglia are independent of the other three microglial subtypes. Therefore, we speculate that *Spp1*^+^ microglia are the source of iBRB cell damage in rats with NPDR. Of course, in addition to cell differentiation, the increase in the proportion of *Spp1*^+^ microglia may be related to the increased apoptosis of the other three subtypes of microglia, or the damaged iBRB environment may be more conducive to the survival of *Spp1*^+^ microglia.

### Promoting oxidative stress and energy metabolism phenotype of microglial cells orchestrates the progression of iBRB injury formation

To further delineate the functional profile of microglial cells and their subtype, Microglia(2), at the outset of the differentiation locus, we conducted gene set variation analysis (GSVA) on single-cell transcriptomic data to investigate the effector pathways responsible for the formation of iBRB injury. Additionally, UCell was employed to calculate the single-cell scores in each pathway, allowing us to explore the spatial distribution of specific pathways.

UCell analysis revealed that, compared to other cell populations, genes related to the AGE-RAGE signaling pathway and apoptosis were significantly enriched in the Microglia population. This indicates that Microglia cells predominantly drive the process of diabetic retinopathy. Furthermore, Microglia cells play a crucial role in angiogenesis and heme metabolism in DR, suggesting their involvement in pathological angiogenesis and remodeling, which in turn affects oxygen transport. Pathways such as IL6/JAK/STAT3 signaling, MTORC1 signaling, inflammatory response, and the P53 pathway were also significantly up-regulated in Microglia (see Supplementary Fig. 5A-B).

**Fig. 5:**
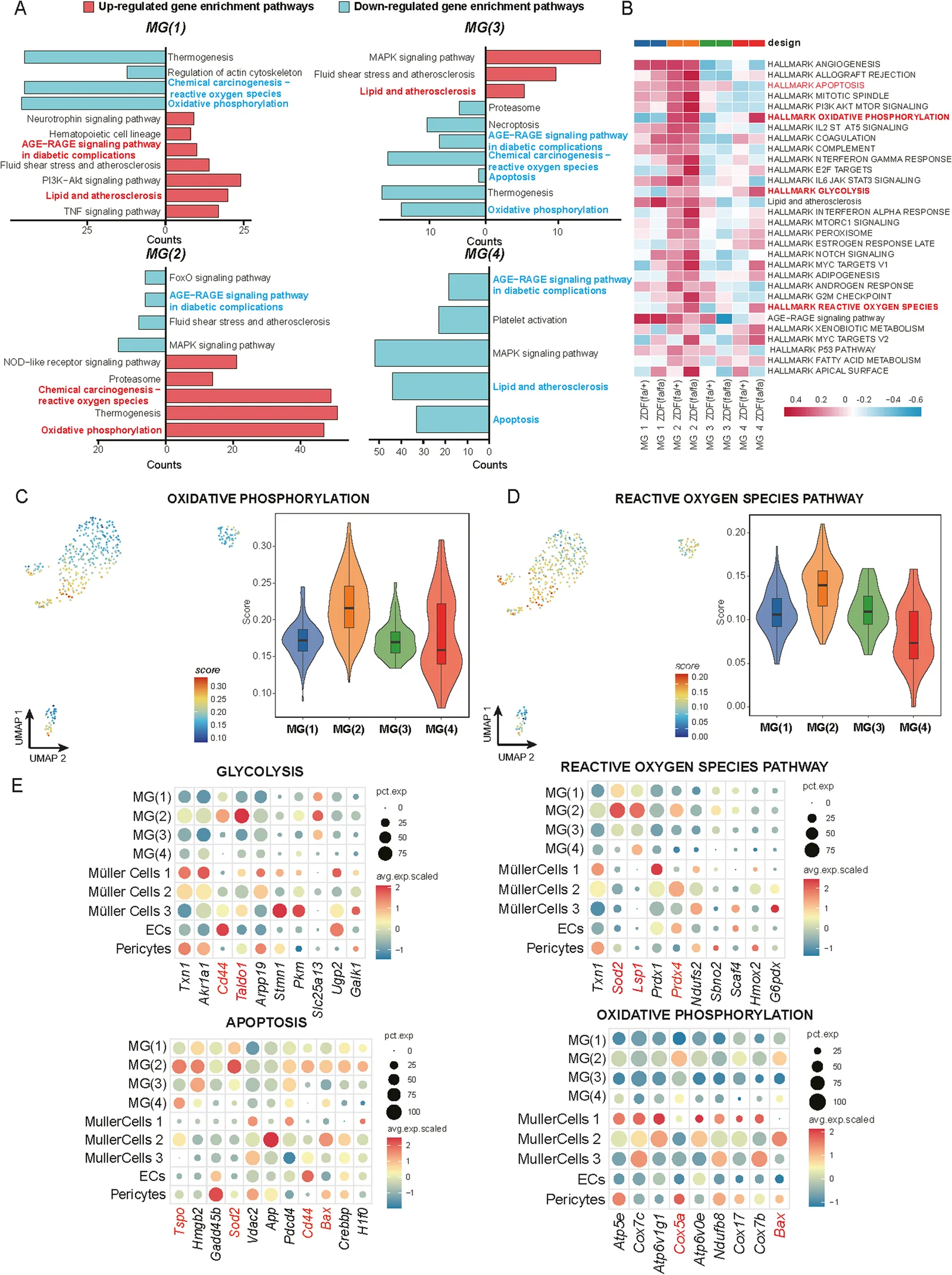
The activated Microglia on ZDF rats’ retinas represent both pro-oxidative phosphorylation and pro-glycolysis properties. **A** KEGG analysis pathway bars for four microglial subsets, the abscissa represents the number of genes enriched in the pathway, green represents the down-regulated pathway and red represents the up-regulated pathway. **B** The scoring matrix corresponds to the top 31 differential pathway heatmap. **C**-**D** UMAP map and box plot of the UCell score in the oxidative phosphorylation (**C**) and reactive oxygen species pathway (**D**) gene sets. **E** The GSVA gene set included the expression of genes involved in glycolysis, reactive oxygen species ROS pathway, apoptosis, and oxidative phosphorylation pathway in each cell subpopulation in the iBRB microenvironment. Abbreviations: MG, microglia

The pro-inflammatory and vascular remodeling effects of the AGE-RAGE signaling pathway are well-documented, indicating that microglia are essential in fostering vascular injury, remodeling, and mediating inflammation in the iBRB microenvironment. KEGG enrichment analysis was also employed to investigate the specific signatures in each Microglia subtype based on their top 11 marker pathways. In comparison with ZDF(fa/+) rats, the down-regulated genes of retinal Microglia (2) in ZDF rats were chiefly enriched in MAPK signaling pathways, flow shear stress, atherosclerosis pathways, autophagy, AGE-RAGE signaling, and FOXO signaling pathways. Conversely, up-regulated genes were predominantly enriched in NOD-like receptor signaling pathways, proteasome, chemical carcinogenesis, reactive oxygen species, thermogenesis, and oxidative phosphorylation pathways, while other microglia subtypes exhibited distinct roles (see Fig. 5A, Supplementary Table 2).

Notably, the gene set associated with oxidative phosphorylation and peroxisome was upregulated in Microglia (2) (see Fig. 5C). Specific genes within the OXPHOS pathways, such as ATP synthase subunit epsilon, mitochondrial (ATP5E), Cytochrome c oxidase subunit 7 C (Cox7c), V-type proton ATPase subunit G 1 (Atp6v1g1), Voltage-dependent anion-selective channel protein 2 (Vdac2), Cytochrome c oxidase subunit 5 A (Cox5a), and V-type proton ATPase subunit e 1 (Atp6v0e), were found abundantly in Microglia (2), potentially contributing to energy metabolism and oxidative stress (see Fig. 5E). Furthermore, more than 20 hallmark pathways were markedly enriched in Microglia (2) in both ZDF(fa/+) and ZDF rats, with gene sets for apoptosis, oxidative phosphorylation, glycolysis, reactive oxygen species, and other pathways being significantly concentrated in Microglia (2) (see Fig. 5B).

In order to further explore the role of Microglia (2) in oxidative phosphorylation and reactive oxygen species, apoptosis, and glycolysis in NPDR retinal microglia in ZDF rats, we explored the genes specifically regulated by Microglia (2) in the data scored by the GSVA gene set. As shown in Fig. 5E, in glycolysis, the expression of many genes in Microglia (2) is higher than that of other microglial subsets, with the highest being *Cd44* and *Talto1*. Among the reactive oxygen species, Sod2, Lsp1, and Prdx4 were most significantly expressed in Microglia (2). In the apoptotic pathway, the expression of *Tspo*, *Sod2*, *Pdcd4*, *Cd44*, and *Bax*, in Microglia (2) was significantly higher than that in other iBRB subsets. In the oxidative phosphorylation pathway, the genes with significant high expression of Microglia (2) are *Cdx5a* and *Bax*.

All in all, Microglia (2) is the group of cells in iBRB that are mainly involved in energy supply and reactive oxygen species production, and participate in and regulate apoptosis and glycolysis.

### The expression level of SPP1 was positively correlated with iBRB injury in ZDF rats with NPDR

**Fig. 6:**
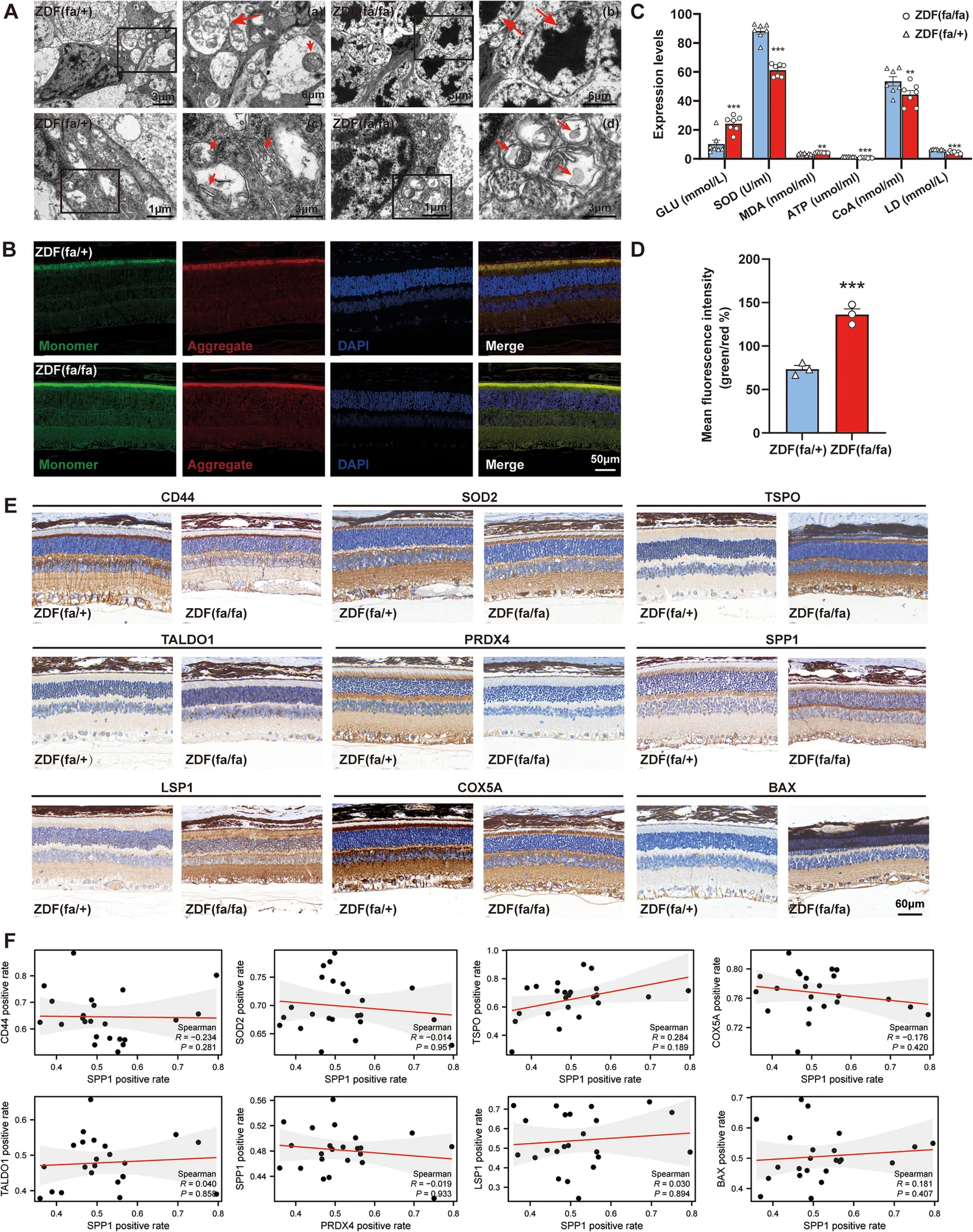
Oxidative stress and apoptosis occur during NPDR lesions. **A** TEM of retinal microglia in ZDF(fa/+) and ZDF(fa/fa) rats. **B**, **D** Retinal JC-1 immunofluorescence staining (**B**) and result statistics (**D**) (*n* = 3). The stronger the green color and the weaker the red, the lower the mitochondrial membrane potential and the more severe the mitochondrial damage. **C** ELISA detection of markers related to glycolysis and oxidative stress in plasma of ZDF rats (*n* = 7). **E**-**F** Immunohistochemical results of CD44, SOD2, TSPO, TALDO1, PRDX4, SPP1, LSP1, COX5A, and BAX in rat retina in the control group and NPDR group (**E**) and their correlation with SPP1 trend plot (**F**). (*P < 0.05, **P < 0.01, ***P < 0.001)

To investigate whether oxidative stress, glycolysis, and apoptosis occur in microglia or other retinal cells during NPDR, and to examine the correlation of SPP1 in these processes, we conducted several experiments. Transmission electron microscopy (TEM) revealed that retinal microglia in the ZDF(fa/+) group exhibited regular morphology and abundant cellular content. In contrast, microglia in the ZDF(fa/fa) group appeared swollen, with lysed chromatin, vascularized cytoplasmic organelles, ruptured membranes, and released cellular contents (Fig. 6A, panels a-b). The retinal mitochondrial membrane potential in the ZDF(fa/+) group on a standard diet remained normal, with mitochondria predominantly long and tubular at the cell body-axon interface and featuring a moderate number of cristae folds, perpendicular to the longitudinal axis of the membrane. In the ZDF(fa/fa) group, decreased membrane potential, significantly impaired mitochondrial structure, predominantly oval shape, low matrix density, reduced or damaged cristae, and incidents of mitochondrial fragmentation, shortening, or high swelling were observed (Fig. 6A, panels c-d; Fig. 6B, D). Concurrently, the ZDF(fa/fa) group exhibited decreased superoxide dismutase levels and a significant increase in the oxidative product MDA compared to the ZDF(fa/+) group, indicating oxidative stress formation.

Moreover, there was a significant increase in blood glucose, an decrease in CoA, and a noticeable decrease in lactate and ATP levels, suggesting decreased anaerobic oxidation and inhibition of the tricarboxylic acid cycle (Fig. 6C). Immunohistochemistry results demonstrated a marked increase in SPP1 expression during NPDR lesions, with CD44, SOD2, PRDX4, and COX5A levels negatively correlating with SPP1, while TSPO, TALDO1, LSP1, and BAX levels positively correlated with SPP1 content (Fig. 6E-F). Interestingly, we also observed a positive association of BAX and TALDO1 with SPP1 and a negative association of SOD2 with SPP1 in the NPDR model of C57BL/6 mice (Supplementary Fig. 6A-B). In summary, oxidative stress and energy metabolism disorders are evident in microglia and other retinal cells during NPDR lesions, ultimately leading to apoptosis and disruption of the iBRB microenvironment. These processes may be related to the generation of an *Spp1*^+^ microglial population.

Similarly, we constructed an *Spp1* knockdown BV2 cell line in vitro, and selected the siRNA with the highest knockdown efficiency for the subsequent experiments (Supplementary Fig. 7A). The results showed that knockdown of Spp1 could significantly reverse the decrease in microglial cell viability caused by LPS (Supplementary Fig. 7B) and the increase in reactive oxygen species (ROS) (Supplementary Figs. 7 C-D). These results indicate that knocking down *Spp1* can enhance the survival rate of microglia and improve oxidative stress.

GEXSCOPE.

## Disscussion

One of the hallmarks of NPDR, an early stage of DR, is the impairment of the iBRB which likely progresses to excessive retinal vascular leakage, eventually reaching advanced stages of DR and causing severe, permanent visual impairment and blindness in adults [32]. Despite recognizing NPDR symptoms, the mechanisms behind cell shedding, tight junction damage, and microvascular injury in the iBRB microenvironment, along with the sequence of events and potential interventions to repair the diseased iBRB network, remain undeveloped [33]. In this study, we analyzed retina samples from normal and NPDR Zucker Diabetic Fatty (ZDF) rats through single-cell RNA sequencing to investigate the pathogenesis and construct the iBRB microenvironment network.

Microglia, as resident retinal cells, are implicated in the relationship between their activation and iBRB disruption [8] Activated microglia produce pro-inflammatory factors, and clinical studies have reported elevated inflammatory factor levels in the vitreous of patients with various ischemic retinal diseases, including DR. Anti-inflammatory treatments like steroids can alleviate retinal edema in DR patients resistant to anti-VEGF therapy [9]. These observations indicate a close relationship between microglia and retinal vascular permeability. Prior research identified novel pericyte types involved in angiogenesis and retinal microvascular / permeability through single-cell sequencing [12]. However, there has been insufficient research on microglia within the iBRB microenvironment. Our findings revealed that the DEGs in endothelial cells, Müller cells, pericytes, and microglia in the iBRB microenvironment were significantly enriched in pathways related to blood flow, diabetes, and cell death (Fig. 2F), with microglia exhibiting the highest connectivity. Furthermore, GSEA analysis in the HALLMARK database showed significant enrichment of DEGs in oxidative phosphorylation pathways in endothelial cells, Müller cells, and microglia (Fig. 2G). Cell-cell interaction analysis by Cellphone DB indicated that microglia had the lowest interaction degree with other cell types in NPDR lesions (Fig. 3D). These results suggest that microglia play an essential role in the iBRB microenvironment of NPDR lesions.

Further, we observed a significant increase in the proportion of microglial population 2, characterized by high Spp1 expression, in ZDF(fa/fa) rats compared to ZDF(fa/+) rats (Fig. 4A-B). In differentiation trajectories analyzed by RNAVelocity and Monocle 2, Microglial (2) was positioned at the differentiation starting point, differentiating into populations 1 and 3 and demonstrating the highest differentiation potential among all microglial subsets in CytoTrace. Thus, we propose that microglial population 2, with elevated Spp1 expression, is a critical contributor to iBRB injury and was the focus of subsequent analyses. Enrichment and GSVA analyses of the four microglial subsets suggested that population 2 was closely linked to oxidative phosphorylation, oxidative stress, glycolysis, and apoptosis (Fig. 5A-D).

To test these hypotheses, ZDF(fa/fa) rats were fed a high-fat diet for 8 weeks. TEM results showed that, compared to ZDF(fa/+), ZDF(fa/fa) rats exhibited swollen mitochondria, cristae loss, decreased cell content, reduced mitochondrial membrane potential, and weakened mitochondrial function (Fig. 6A-B, D). Superoxide dismutase (SOD), an antioxidant defense enzyme [34], was significantly decreased in NPDR group plasma, while malondialdehyde (MDA), a recognized oxidative stress product [35], was significantly increased. Plasma glucose was elevated, whereas glycolytic products such as acetyl-CoA and lactate were decreased, indicating inhibited glycolysis in NPDR rats (Fig. 6C). The ROS-related protein LSP1 was positively correlated with SPP1 expression, while the expression of the antioxidant protein PRDX4 [36] was negatively correlated. Glycolysis-related protein CD44, produced by aerobic glycolysis [37], decreased, whereas the expression of TALDO1, a pentose phosphate pathway rate-limiting enzyme [38], increased. TSPO, a high-affinity cholesterol-binding protein, is involved in apoptosis, and TSPO knockout reduces cell apoptosis [39]. BAX, a key mitochondrial cell death executor [40] was upregulated in the NPDR retina and positively correlated with SPP1 expression. These observations suggest that SPP1 overexpression in Microglia (2) is associated with oxidative stress, glycolysis inhibition, and apoptosis.

*Spp1* ^+^ microglia have been documented in numerous studies. Through single-cell RNA sequencing analysis, Wang et al. revealed that SPP1 is associated with the activation and proliferation of microglia [41]. Furthermore, several studies have indicated that Spp1-positive microglia exhibit enhanced phagocytic activity, whereas *Ccl13*^*+*^ microglia show significantly reduced phagocytosis [42]. Alterations in microglial phagocytosis and complement activation mediated by SPP1 may also contribute to the regulation of synaptic pruning in mice [43]. During neonatal or juvenile stroke, *Spp1*^*+*^ microglia serve as a source of disease-associated microglia in an irreversible state [41], potentially linked to inflammation and cellular injury—a finding consistent with our own results.

**Fig. 7:**
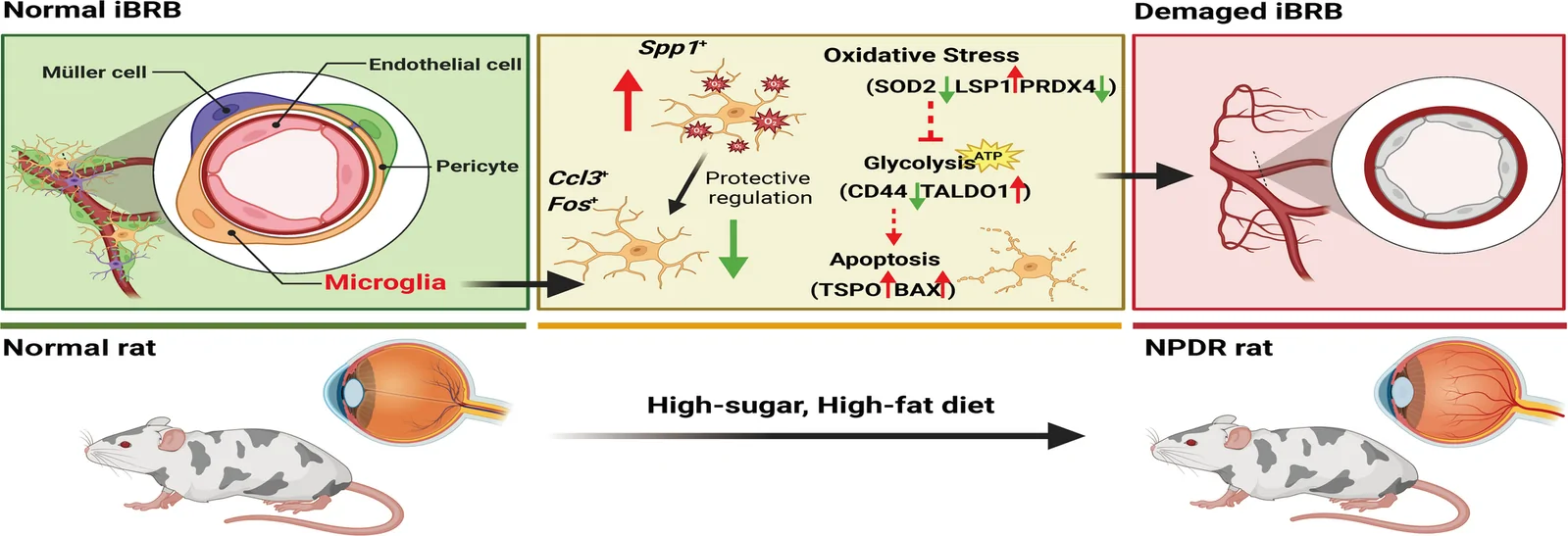
Graphical abstract of the article. In the normal rat retina, the inner blood-retinal barrier is composed of tight junctions such as Muller cells, endothelial cells, peridermal cells, and microglia to protect the normal function of the retina. After rats were fed a high-sugar and high-fat diet, the proportion of the *Ccl3*^+^ and the *Fos*^+^ microglia populations in iBRB decreased. The proportion of the *Spp1*^+^ microglia population increased, while a portion of the protective regulation converted to the *Ccl3* + and the *Fos*^+^ microglia. The process of oxidative stress in the *Spp1*^+^ microglia is increased, the glycolytic process is inhibited, and apoptosis is increased. Further, the cellular connections in the iBRB microenvironment are disrupted, and the iBRB is damaged, which leads to the development of diabetic retinopathy

Advancements in scRNA-seq with computational tools are enabling the identification of disease subtypes and mechanisms [44], offering a powerful method to define cellular heterogeneity under pathological conditions [45]. In this study, summarized in Fig. 7, we employed scRNA-seq combined with bioinformatics to explore relationships within the iBRB microenvironment. In normal rat retinas, the iBRB comprises tight junctions involving Müller cells, endothelial cells, epithelial cells, and microglia, preserving retinal function. After a high-sugar, high-fat diet, the Ccl3^+^ and Fos^+^ microglia populations in the iBRB were reduced, whereas Spp1^+^ microglia increased, converting part of the protective mechanism to Ccl3^+^ and Fos^+^ microglia. Notably, oxidative stress intensified, glycolysis was inhibited, and apoptosis increased in Spp1^+^ microglia. Resultingly, cell junction disruption in the iBRB microenvironment led to diabetic retinopathy progression. SPP1 and associated regulatory proteins were validated in vitro. This study, therefore, delineates the implications of microglial heterogeneity on iBRB impairment at a single-cell level.

Nevertheless, our study has limitations despite providing a rationale. Therefore, future research employing conditional knockout rat models is critical to gain a more definitive understanding of the biological mechanisms in vivo. However, integrating the single-cell transcriptomic data generated in this study with proteomic or metabolomic analyses in the future would be particularly valuable.

## Conclusion

In summary, we constructed a single-cell transcriptome atlas for NPDR, focusing on the inner blood-retinal barrier, particularly microglia, through various analytical methods, revealing heterogeneity in functional enrichment, cell differentiation trajectories, and intercellular communication. A distinctive population of Spp1^+^ microglia with high differentiation potential was identified as an origin of differentiation, with a significantly increased presence in diseased retinae, potentially linked to oxidative stress, glycolysis inhibition, and apoptosis in microglia. In vivo studies confirmed SPP1’s involvement. Our research provides an accurate reference for developing targeted therapies for NPDR patients and deepens the understanding of microglial regulation in NPDR. However, further validation through experimental and clinical studies is necessary.

## Supplementary Information


Supplementary Material 1.


## Data Availability

The raw data of scRNA-seq generated in this study were deposited in Genome Sequence Archive40 in National Genomics Data Center, China National Center for Bioinformation/Beijing Institute of Genomics, Chinese Academy of Sciences (CRA033021) that are publicly accessible at https://ngdc.cncb.ac.cn/gsa.

## Abbreviations

DR: Diabetic retinopathy
NPDR: Non-proliferative diabetic retinopathy
PDR: Proliferative diabetic retinopathy
iBRB: Inner blood-retinal barrier
scRNA-seq: Single-cell RNA sequencing
T2D: Type 2 diabetes
RLBP1: RalA Binding Protein 1
ZDF: Zucker diabetic fat
UMIs: Unique molecular identifiers
UMAP: Uniform Manifold Approximation and Projection
DEGs: Differentially expressed genes
UREA: Urea
CRE: Crea
LDH: Llactatedehydrogenase
CK-MB: Creatine Kinase-MB
ALT: Alanine aminotransferase
AST: Aspartate transaminase
ELISA: Enzyme linked immunosorbent assay
SOD: Superoxide dismutas
ATP: Adenosine triphosphate
MDA: Malonaldehyde
CoA: Acetyl-CoA
FFA: Fundus fluorescein angiography
HE stain: Hematoxylin-eosin staining
PCA: Principle Component Analysis
nFeature: Gene number in each cell
nCount: Total count number of all genes in each cel
percent.m: Percentage of mitochondrial gene number in total gene number in each cell
t-SNE: t-distributed Stochastic Neighbor Embedding
GSEA: Gene Set Enrichment Analysis
ROS: Reactive oxygen species
MG: Microglia
SPP1: Sphingosine-1-phosphate phosphatase 1
BAX: Apoptosis regulator BAX
TALDO1: Transaldolase
LSP1: Lymphocyte-specific protein 1
SOD2: Superoxide dismutase [Mn], mitochondrial
PRDX4: Peroxiredoxin-4
TSPO: Translocator protei
COX5A: Cytochrome c oxidase subunit 5A
