# Supplementary material for: Unveiling a unique microglial phenotype promoting oxidation in the iBRB: insights from single-cell transcriptomics in the NPDR rat model
Source: Cell Biosci. 2026 Jun 19;16:101. doi: 10.1186/s13578-026-01613-z (PMC13523196; doi:10.1186/s13578-026-01613-z)
Supplement: Supplementary file 1 [file 13578_2026_1613_MOESM1_ESM.docx]

**Supplementary Figure 1.** Indicators for establishing NPDR models in ZDF rats**.** (A) Contents of LDH and CK-MB in the hearts of ZDF(fa/+) and ZDF(fa/fa) rats (n=5). (B) Contents of ALT and AST in the livers of ZDF(fa/+) and ZDF(fa/fa) rats (n=5). Abbreviations: LDH, Lactate Dehydrogenase; CK-MB, Creatine Kinase-MB; ALT, Alanine aminotransferase; AST, Aspartate Aminotransferase. (**P* < 0.05, ***P* < 0.01)

**Supplementary Figure 2.** UMAP representation of single-cell gene expression in every sample. (A-F) UMAP plots of the 9 cell types in each sample.

**Supplementary Figure 3.** Immunofluorescence staining of *Spp1*⁺ microglia and three other microglial subtypes in NPDR rats. (A) Localization of *q* in the retina of NPDR rats with *Fos*^+^ microglia, *Cxcl13*^+^ microglia and *Ccl3*^+^ microglia, respectively. (IBA1: Ionized calcium-binding adapter molecule 1, the marker of Microglial cells）

**Supplementary Figure 4.** Clustered heatmap of the AUC matrix in each of the four cell types from ZDF(fa/+) and ZDF(fa/fa) rats (A) .

**Supplementary Figure 5.** Marker genes for each cell type in the retina. (A) Violin plots show the differential genes for Top3 for each cell type in the retina. (B) Violin plot of pathways in which UCell scores microglia line the front in the Hallmark database.

**Supplementary Figure 6.** The correlation analysis between SPP1 and oxidative stress, apoptosis and other pathways in the NPDR model of C57BL/6 mice. (A) Immunohistochemical staining of SPP1, SOD2, BAX and TALDO1 in the retina of Control and NPDR mice (n=4). (B) Dot plot of correlation between SPP1 and BAX, TALDO1, and SOD2 positive rates.

**Supplementary Figure 7.** Knockdown of Spp1 improves the survival rate of microglia and reduces oxidative stress in the BV2 cell line. (A) After transfection with three siRNA sequences, the gene expression level of Spp1 was measured. Select sequence 3 for the subsequent experiments. (B) Cell survival rate after 24 hours of stimulation with 1 μg/ml LPS. (C-D) The fluorescence intensity of reactive oxygen species (ROS) in the Spp1 knockdown group and the control group after LPS stimulation.

**Supplementary Table 1. The KEGG analysis enrichment pathways of the four types of cells in iBRB**

| Cell type | ID | Description | p.adjust |
| --- | --- | --- | --- |
| ECs | mmu04260 | Cardiac muscle contraction | 0.0005 |
| ECs | mmu03018 | RNA degradation | 0.0008 |
| ECs | mmu04137 | Mitophagy - animal | 0.0011 |
| ECs | mmu04120 | Ubiquitin mediated proteolysis | 0.0014 |
| ECs | mmu00020 | Citrate cycle (TCA cycle) | 0.0020 |
| ECs | mmu04066 | HIF-1 signaling pathway | 0.0037 |
| ECs | mmu05225 | Hepatocellular carcinoma | 0.0002 |
| ECs | mmu04928 | Parathyroid hormone synthesis, secretion and action | 0.0003 |
| ECs | mmu04971 | Gastric acid secretion | 0.0005 |
| ECs | mmu04015 | Rap1 signaling pathway | 0.0006 |
| ECs | mmu05224 | Breast cancer | 0.0006 |
| ECs | mmu04724 | Glutamatergic synapse | 0.0013 |
| ECs | mmu04713 | Circadian entrainment | 0.0013 |
| ECs | mmu05132 | Salmonella infection | 0.0014 |
| ECs | mmu04933 | AGE-RAGE signaling pathway in diabetic complications | 0.0018 |
| ECs | mmu04915 | Estrogen signaling pathway | 0.0018 |
| ECs | mmu04919 | Thyroid hormone signaling pathway | 0.0024 |
| ECs | mmu04926 | Relaxin signaling pathway | 0.0024 |
| ECs | mmu04330 | Notch signaling pathway | 0.0027 |
| ECs | mmu05032 | Morphine addiction | 0.0029 |
| ECs | mmu05100 | Bacterial invasion of epithelial cells | 0.0029 |
| ECs | mmu04611 | Platelet activation | 0.0029 |
| ECs | mmu04014 | Ras signaling pathway | 0.0032 |
| ECs | mmu05222 | Small cell lung cancer | 0.0033 |
| ECs | mmu04923 | Regulation of lipolysis in adipocytes | 0.0036 |
| ECs | mmu04520 | Adherens junction | 0.0037 |
| ECs | mmu04024 | cAMP signaling pathway | 0.0039 |
| ECs | mmu05163 | Human cytomegalovirus infection | 0.0039 |
| ECs | mmu05223 | Non-small cell lung cancer | 0.0039 |
| ECs | mmu04727 | GABAergic synapse | 0.0046 |
| ECs | mmu04010 | MAPK signaling pathway | 0.0067 |
| ECs | mmu04728 | Dopaminergic synapse | 0.0068 |
| ECs | mmu04934 | Cushing syndrome | 0.0068 |
| ECs | mmu04810 | Regulation of actin cytoskeleton | 0.0079 |
| ECs | mmu04390 | Hippo signaling pathway | 0.0092 |
| ECs | mmu05165 | Human papillomavirus infection | 0.0093 |
| ECs | mmu04142 | Lysosome | 0.0096 |
| ECs | mmu05207 | Chemical carcinogenesis - receptor activation | 0.0102 |
| ECs | mmu04340 | Hedgehog signaling pathway | 0.0116 |
| ECs | mmu04918 | Thyroid hormone synthesis | 0.0118 |
| ECs | mmu04974 | Protein digestion and absorption | 0.0121 |
| ECs | mmu04921 | Oxytocin signaling pathway | 0.0122 |
| ECs | mmu04210 | Apoptosis | 0.0133 |
| ECs | mmu04730 | Long-term depression | 0.0139 |
| ECs | mmu04371 | Apelin signaling pathway | 0.0139 |
| ECs | mmu04144 | Endocytosis | 0.0148 |
| ECs | mmu05412 | Arrhythmogenic right ventricular cardiomyopathy | 0.0148 |
| ECs | mmu04725 | Cholinergic synapse | 0.0157 |
| ECs | mmu05226 | Gastric cancer | 0.0175 |
| ECs | mmu04151 | PI3K-Akt signaling pathway | 0.0175 |
| ECs | mmu05217 | Basal cell carcinoma | 0.0182 |
| ECs | mmu05210 | Colorectal cancer | 0.0182 |
| ECs | mmu05218 | Melanoma | 0.0198 |
| ECs | mmu05170 | Human immunodeficiency virus 1 infection | 0.0199 |
| ECs | mmu04514 | Cell adhesion molecules | 0.0235 |
| ECs | mmu04670 | Leukocyte transendothelial migration | 0.0235 |
| ECs | mmu04916 | Melanogenesis | 0.0235 |
| ECs | mmu04530 | Tight junction | 0.0272 |
| ECs | mmu04924 | Renin secretion | 0.0287 |
| ECs | mmu04140 | Autophagy - animal | 0.0292 |
| ECs | mmu04550 | Signaling pathways regulating pluripotency of stem cells | 0.0292 |
| ECs | mmu03060 | Protein export | 0.0292 |
| ECs | mmu04668 | TNF signaling pathway | 0.0305 |
| ECs | mmu04540 | Gap junction | 0.0305 |
| ECs | mmu04261 | Adrenergic signaling in cardiomyocytes | 0.0334 |
| ECs | mmu04071 | Sphingolipid signaling pathway | 0.0342 |
| ECs | mmu05167 | Kaposi sarcoma-associated herpesvirus infection | 0.0384 |
| ECs | mmu04218 | Cellular senescence | 0.0384 |
| ECs | mmu00480 | Glutathione metabolism | 0.0411 |
| ECs | mmu05215 | Prostate cancer | 0.0411 |
| ECs | mmu04612 | Antigen processing and presentation | 0.0411 |
| ECs | mmu05030 | Cocaine addiction | 0.0454 |
| ECs | mmu04216 | Ferroptosis | 0.0454 |
| ECs | mmu04310 | Wnt signaling pathway | 0.0454 |
| ECs | mmu05214 | Glioma | 0.0469 |
| ECs | mmu01522 | Endocrine resistance | 0.0507 |
| ECs | mmu04726 | Serotonergic synapse | 0.0508 |
| ECs | mmu04625 | C-type lectin receptor signaling pathway | 0.0508 |
| ECs | mmu05414 | Dilated cardiomyopathy | 0.0534 |
| ECs | mmu05212 | Pancreatic cancer | 0.0536 |
| ECs | mmu00600 | Sphingolipid metabolism | 0.0536 |
| ECs | mmu04350 | TGF-beta signaling pathway | 0.0559 |
| ECs | mmu04022 | cGMP-PKG signaling pathway | 0.0559 |
| ECs | mmu05211 | Renal cell carcinoma | 0.0560 |
| ECs | mmu05133 | Pertussis | 0.0560 |
| ECs | mmu05135 | Yersinia infection | 0.0567 |
| ECs | mmu04935 | Growth hormone synthesis, secretion and action | 0.0623 |
| ECs | mmu04512 | ECM-receptor interaction | 0.0640 |
| ECs | mmu04723 | Retrograde endocannabinoid signaling | 0.0722 |
| ECs | mmu04912 | GnRH signaling pathway | 0.0743 |
| ECs | mmu05010 | Alzheimer disease | 0.0748 |
| ECs | mmu05410 | Hypertrophic cardiomyopathy | 0.0790 |
| ECs | mmu04964 | Proximal tubule bicarbonate reclamation | 0.0822 |
| ECs | mmu04722 | Neurotrophin signaling pathway | 0.0827 |
| ECs | mmu04145 | Phagosome | 0.0827 |
| ECs | mmu04960 | Aldosterone-regulated sodium reabsorption | 0.0827 |
| ECs | mmu04925 | Aldosterone synthesis and secretion | 0.0837 |
| ECs | mmu05206 | MicroRNAs in cancer | 0.0942 |
| ECs | mmu00640 | Propanoate metabolism | 0.0942 |
| ECs | mmu04922 | Glucagon signaling pathway | 0.0942 |
| ECs | mmu04066 | HIF-1 signaling pathway | 0.0942 |
| ECs | mmu00260 | Glycine, serine and threonine metabolism | 0.1001 |
| ECs | mmu04970 | Salivary secretion | 0.1001 |
| ECs | mmu04979 | Cholesterol metabolism | 0.1001 |
| ECs | mmu04370 | VEGF signaling pathway | 0.1001 |
| ECs | mmu05213 | Endometrial cancer | 0.1001 |
| ECs | mmu04137 | Mitophagy - animal | 0.1065 |
| ECs | mmu05219 | Bladder cancer | 0.1071 |
| ECs | mmu00510 | N-Glycan biosynthesis | 0.1072 |
| ECs | mmu02010 | ABC transporters | 0.1072 |
| ECs | mmu00910 | Nitrogen metabolism | 0.1081 |
| ECs | mmu05230 | Central carbon metabolism in cancer | 0.1110 |
| ECs | mmu01230 | Biosynthesis of amino acids | 0.1143 |
| ECs | mmu01524 | Platinum drug resistance | 0.1225 |
| ECs | mmu00511 | Other glycan degradation | 0.1261 |
| ECs | mmu01200 | Carbon metabolism | 0.1291 |
| Microglia Cells | mmu03018 | RNA degradation | 0.0002 |
| Microglia Cells | mmu04012 | ErbB signaling pathway | 0.0002 |
| Microglia Cells | mmu04728 | Dopaminergic synapse | 0.0003 |
| Microglia Cells | mmu01230 | Biosynthesis of amino acids | 0.0003 |
| Microglia Cells | mmu00020 | Citrate cycle (TCA cycle) | 0.0004 |
| Microglia Cells | mmu04120 | Ubiquitin mediated proteolysis | 0.0005 |
| Microglia Cells | mmu04721 | Synaptic vesicle cycle | 0.0005 |
| Microglia Cells | mmu00010 | Glycolysis / Gluconeogenesis | 0.0008 |
| Microglia Cells | mmu04360 | Axon guidance | 0.0009 |
| Microglia Cells | mmu05230 | Central carbon metabolism in cancer | 0.0011 |
| Microglia Cells | mmu04727 | GABAergic synapse | 0.0014 |
| Microglia Cells | mmu01210 | 2-Oxocarboxylic acid metabolism | 0.0042 |
| Microglia Cells | mmu04520 | Adherens junction | 0.0046 |
| Microglia Cells | mmu05231 | Choline metabolism in cancer | 0.0050 |
| Microglia Cells | mmu05213 | Endometrial cancer | 0.0123 |
| Microglia Cells | mmu04141 | Protein processing in endoplasmic reticulum | 0.0136 |
| Microglia Cells | mmu01522 | Endocrine resistance | 0.0152 |
| Microglia Cells | mmu04710 | Circadian rhythm | 0.0166 |
| Microglia Cells | mmu04930 | Type II diabetes mellitus | 0.0174 |
| Microglia Cells | mmu05211 | Renal cell carcinoma | 0.0189 |
| Microglia Cells | mmu04931 | Insulin resistance | 0.0189 |
| Microglia Cells | mmu04070 | Phosphatidylinositol signaling system | 0.0189 |
| Microglia Cells | mmu04144 | Endocytosis | 0.0189 |
| Microglia Cells | mmu04140 | Autophagy - animal | 0.0189 |
| Microglia Cells | mmu03013 | Nucleocytoplasmic transport | 0.0189 |
| Microglia Cells | mmu04922 | Glucagon signaling pathway | 0.0192 |
| Microglia Cells | mmu04150 | mTOR signaling pathway | 0.0192 |
| Microglia Cells | mmu04713 | Circadian entrainment | 0.0214 |
| Microglia Cells | mmu04724 | Glutamatergic synapse | 0.0220 |
| Microglia Cells | mmu00310 | Lysine degradation | 0.0220 |
| Microglia Cells | mmu04722 | Neurotrophin signaling pathway | 0.0220 |
| Microglia Cells | mmu04666 | Fc gamma R-mediated phagocytosis | 0.0220 |
| Microglia Cells | mmu04914 | Progesterone-mediated oocyte maturation | 0.0220 |
| Microglia Cells | mmu04964 | Proximal tubule bicarbonate reclamation | 0.0252 |
| Microglia Cells | mmu05210 | Colorectal cancer | 0.0307 |
| Microglia Cells | mmu04720 | Long-term potentiation | 0.0307 |
| Microglia Cells | mmu03015 | mRNA surveillance pathway | 0.0314 |
| Microglia Cells | mmu05132 | Salmonella infection | 0.0324 |
| Microglia Cells | mmu04725 | Cholinergic synapse | 0.0356 |
| Microglia Cells | mmu04211 | Longevity regulating pathway | 0.0356 |
| Microglia Cells | mmu04919 | Thyroid hormone signaling pathway | 0.0361 |
| Microglia Cells | mmu04114 | Oocyte meiosis | 0.0390 |
| Microglia Cells | mmu04926 | Relaxin signaling pathway | 0.0397 |
| Microglia Cells | mmu04910 | Insulin signaling pathway | 0.0465 |
| Microglia Cells | mmu00620 | Pyruvate metabolism | 0.0465 |
| Microglia Cells | mmu04962 | Vasopressin-regulated water reabsorption | 0.0465 |
| Microglia Cells | mmu04071 | Sphingolipid signaling pathway | 0.0468 |
| Microglia Cells | mmu01521 | EGFR tyrosine kinase inhibitor resistance | 0.0468 |
| Microglia Cells | mmu04370 | VEGF signaling pathway | 0.0476 |
| Microglia Cells | mmu04960 | Aldosterone-regulated sodium reabsorption | 0.0476 |
| Microglia Cells | mmu04660 | T cell receptor signaling pathway | 0.0536 |
| Microglia Cells | mmu05214 | Glioma | 0.0573 |
| Microglia Cells | mmu04137 | Mitophagy - animal | 0.0654 |
| Microglia Cells | mmu04928 | Parathyroid hormone synthesis, secretion and action | 0.0001 |
| Microglia Cells | mmu05161 | Hepatitis B | 0.0001 |
| Microglia Cells | mmu05142 | Chagas disease | 0.0001 |
| Microglia Cells | mmu05170 | Human immunodeficiency virus 1 infection | 0.0001 |
| Microglia Cells | mmu05164 | Influenza A | 0.0001 |
| Microglia Cells | mmu05166 | Human T-cell leukemia virus 1 infection | 0.0002 |
| Microglia Cells | mmu04137 | Mitophagy - animal | 0.0002 |
| Microglia Cells | mmu05321 | Inflammatory bowel disease | 0.0002 |
| Microglia Cells | mmu04620 | Toll-like receptor signaling pathway | 0.0002 |
| Microglia Cells | mmu04216 | Ferroptosis | 0.0002 |
| Microglia Cells | mmu04014 | Ras signaling pathway | 0.0003 |
| Microglia Cells | mmu04612 | Antigen processing and presentation | 0.0003 |
| Microglia Cells | mmu05203 | Viral carcinogenesis | 0.0004 |
| Microglia Cells | mmu04151 | PI3K-Akt signaling pathway | 0.0005 |
| Microglia Cells | mmu04650 | Natural killer cell mediated cytotoxicity | 0.0006 |
| Microglia Cells | mmu05211 | Renal cell carcinoma | 0.0006 |
| Microglia Cells | mmu04371 | Apelin signaling pathway | 0.0007 |
| Microglia Cells | mmu04071 | Sphingolipid signaling pathway | 0.0008 |
| Microglia Cells | mmu04657 | IL-17 signaling pathway | 0.0011 |
| Microglia Cells | mmu04072 | Phospholipase D signaling pathway | 0.0012 |
| Microglia Cells | mmu01521 | EGFR tyrosine kinase inhibitor resistance | 0.0013 |
| Microglia Cells | mmu05235 | PD-L1 expression and PD-1 checkpoint pathway in cancer | 0.0019 |
| Microglia Cells | mmu04218 | Cellular senescence | 0.0019 |
| Microglia Cells | mmu04068 | FoxO signaling pathway | 0.0019 |
| Microglia Cells | mmu00511 | Other glycan degradation | 0.0033 |
| Microglia Cells | mmu04926 | Relaxin signaling pathway | 0.0035 |
| Microglia Cells | mmu04115 | p53 signaling pathway | 0.0035 |
| Microglia Cells | mmu05222 | Small cell lung cancer | 0.0036 |
| Microglia Cells | mmu04921 | Oxytocin signaling pathway | 0.0038 |
| Microglia Cells | mmu04640 | Hematopoietic cell lineage | 0.0040 |
| Microglia Cells | mmu05162 | Measles | 0.0040 |
| Microglia Cells | mmu04330 | Notch signaling pathway | 0.0041 |
| Microglia Cells | mmu05214 | Glioma | 0.0044 |
| Microglia Cells | mmu04360 | Axon guidance | 0.0055 |
| Microglia Cells | mmu04217 | Necroptosis | 0.0059 |
| Microglia Cells | mmu05216 | Thyroid cancer | 0.0061 |
| Microglia Cells | mmu05231 | Choline metabolism in cancer | 0.0061 |
| Microglia Cells | mmu05215 | Prostate cancer | 0.0069 |
| Microglia Cells | mmu05223 | Non-small cell lung cancer | 0.0086 |
| Microglia Cells | mmu04140 | Autophagy - animal | 0.0093 |
| Microglia Cells | mmu04730 | Long-term depression | 0.0111 |
| Microglia Cells | mmu01522 | Endocrine resistance | 0.0185 |
| Microglia Cells | mmu05165 | Human papillomavirus infection | 0.0185 |
| Microglia Cells | mmu04540 | Gap junction | 0.0193 |
| Microglia Cells | mmu05144 | Malaria | 0.0193 |
| Microglia Cells | mmu05218 | Melanoma | 0.0209 |
| Microglia Cells | mmu04370 | VEGF signaling pathway | 0.0216 |
| Microglia Cells | mmu05213 | Endometrial cancer | 0.0216 |
| Microglia Cells | mmu04919 | Thyroid hormone signaling pathway | 0.0251 |
| Microglia Cells | mmu04912 | GnRH signaling pathway | 0.0284 |
| Microglia Cells | mmu04971 | Gastric acid secretion | 0.0287 |
| Microglia Cells | mmu04613 | Neutrophil extracellular trap formation | 0.0290 |
| Microglia Cells | mmu00513 | Various types of N-glycan biosynthesis | 0.0315 |
| Microglia Cells | mmu05160 | Hepatitis C | 0.0334 |
| Microglia Cells | mmu04390 | Hippo signaling pathway | 0.0340 |
| Microglia Cells | mmu04915 | Estrogen signaling pathway | 0.0390 |
| Microglia Cells | mmu05414 | Dilated cardiomyopathy | 0.0398 |
| Microglia Cells | mmu04920 | Adipocytokine signaling pathway | 0.0403 |
| Microglia Cells | mmu04061 | Viral protein interaction with cytokine and cytokine receptor | 0.0430 |
| Microglia Cells | mmu04725 | Cholinergic synapse | 0.0464 |
| Müller Cells | mmu03430 | Mismatch repair | 0.9896 |
| Müller Cells | mmu00760 | Nicotinate and nicotinamide metabolism | 0.9896 |
| Müller Cells | mmu03440 | Homologous recombination | 0.9896 |
| Müller Cells | mmu05022 | Pathways of neurodegeneration - multiple diseases | 0.0002 |
| Müller Cells | mmu04721 | Synaptic vesicle cycle | 0.0002 |
| Müller Cells | mmu03013 | Nucleocytoplasmic transport | 0.0003 |
| Müller Cells | mmu04137 | Mitophagy - animal | 0.0003 |
| Müller Cells | mmu05017 | Spinocerebellar ataxia | 0.0005 |
| Müller Cells | mmu05014 | Amyotrophic lateral sclerosis | 0.0005 |
| Müller Cells | mmu04919 | Thyroid hormone signaling pathway | 0.0008 |
| Müller Cells | mmu05132 | Salmonella infection | 0.0009 |
| Müller Cells | mmu04120 | Ubiquitin mediated proteolysis | 0.0009 |
| Müller Cells | mmu05016 | Huntington disease | 0.0009 |
| Müller Cells | mmu03018 | RNA degradation | 0.0009 |
| Müller Cells | mmu05020 | Prion disease | 0.0011 |
| Müller Cells | mmu00310 | Lysine degradation | 0.0011 |
| Müller Cells | mmu04010 | MAPK signaling pathway | 0.0016 |
| Müller Cells | mmu04720 | Long-term potentiation | 0.0018 |
| Müller Cells | mmu04022 | cGMP-PKG signaling pathway | 0.0037 |
| Müller Cells | mmu04922 | Glucagon signaling pathway | 0.0038 |
| Müller Cells | mmu04066 | HIF-1 signaling pathway | 0.0044 |
| Müller Cells | mmu04960 | Aldosterone-regulated sodium reabsorption | 0.0051 |
| Müller Cells | mmu05215 | Prostate cancer | 0.0051 |
| Müller Cells | mmu05211 | Renal cell carcinoma | 0.0056 |
| Müller Cells | mmu04012 | ErbB signaling pathway | 0.0056 |
| Müller Cells | mmu05230 | Central carbon metabolism in cancer | 0.0062 |
| Müller Cells | mmu05208 | Chemical carcinogenesis - reactive oxygen species | 0.0070 |
| Müller Cells | mmu05231 | Choline metabolism in cancer | 0.0104 |
| Müller Cells | mmu04211 | Longevity regulating pathway | 0.0107 |
| Müller Cells | mmu05213 | Endometrial cancer | 0.0110 |
| Müller Cells | mmu05415 | Diabetic cardiomyopathy | 0.0117 |
| Müller Cells | mmu04710 | Circadian rhythm | 0.0120 |
| Müller Cells | mmu04962 | Vasopressin-regulated water reabsorption | 0.0120 |
| Müller Cells | mmu04931 | Insulin resistance | 0.0125 |
| Müller Cells | mmu01522 | Endocrine resistance | 0.0128 |
| Müller Cells | mmu04910 | Insulin signaling pathway | 0.0139 |
| Müller Cells | mmu04660 | T cell receptor signaling pathway | 0.0139 |
| Müller Cells | mmu04114 | Oocyte meiosis | 0.0139 |
| Müller Cells | mmu01521 | EGFR tyrosine kinase inhibitor resistance | 0.0166 |
| Müller Cells | mmu05210 | Colorectal cancer | 0.0167 |
| Müller Cells | mmu05235 | PD-L1 expression and PD-1 checkpoint pathway in cancer | 0.0167 |
| Müller Cells | mmu04714 | Thermogenesis | 0.0176 |
| Müller Cells | mmu04141 | Protein processing in endoplasmic reticulum | 0.0176 |
| Müller Cells | mmu04144 | Endocytosis | 0.0183 |
| Müller Cells | mmu04728 | Dopaminergic synapse | 0.0184 |
| Müller Cells | mmu04932 | Non-alcoholic fatty liver disease | 0.0213 |
| Müller Cells | mmu05417 | Lipid and atherosclerosis | 0.0222 |
| Müller Cells | mmu04722 | Neurotrophin signaling pathway | 0.0259 |
| Müller Cells | mmu04140 | Autophagy - animal | 0.0259 |
| Müller Cells | mmu05170 | Human immunodeficiency virus 1 infection | 0.0259 |
| Müller Cells | mmu04068 | FoxO signaling pathway | 0.0259 |
| Müller Cells | mmu00620 | Pyruvate metabolism | 0.0280 |
| Müller Cells | mmu05012 | Parkinson disease | 0.0300 |
| Müller Cells | mmu04071 | Sphingolipid signaling pathway | 0.0300 |
| Müller Cells | mmu04921 | Oxytocin signaling pathway | 0.0300 |
| Müller Cells | mmu05225 | Hepatocellular carcinoma | 0.0323 |
| Müller Cells | mmu04727 | GABAergic synapse | 0.0348 |
| Müller Cells | mmu04912 | GnRH signaling pathway | 0.0378 |
| Müller Cells | mmu04310 | Wnt signaling pathway | 0.0405 |
| Müller Cells | mmu00020 | Citrate cycle (TCA cycle) | 0.0427 |
| Müller Cells | mmu04914 | Progesterone-mediated oocyte maturation | 0.0436 |
| Müller Cells | mmu05214 | Glioma | 0.0436 |
| Müller Cells | mmu04724 | Glutamatergic synapse | 0.0504 |
| Müller Cells | mmu04370 | VEGF signaling pathway | 0.0504 |
| Müller Cells | mmu05220 | Chronic myeloid leukemia | 0.0509 |
| Müller Cells | mmu05161 | Hepatitis B | 0.0509 |
| Müller Cells | mmu04218 | Cellular senescence | 0.0522 |
| Müller Cells | mmu04070 | Phosphatidylinositol signaling system | 0.0562 |
| Müller Cells | mmu04935 | Growth hormone synthesis, secretion and action | 0.0586 |
| Müller Cells | mmu04150 | mTOR signaling pathway | 0.0586 |
| Müller Cells | mmu04210 | Apoptosis | 0.0586 |
| Müller Cells | mmu03022 | Basal transcription factors | 0.0656 |
| Müller Cells | mmu04520 | Adherens junction | 0.0689 |
| Müller Cells | mmu04360 | Axon guidance | 0.0017 |
| Müller Cells | mmu04371 | Apelin signaling pathway | 0.0026 |
| Müller Cells | mmu04216 | Ferroptosis | 0.0026 |
| Müller Cells | mmu04926 | Relaxin signaling pathway | 0.0026 |
| Müller Cells | mmu05012 | Parkinson disease | 0.0026 |
| Müller Cells | mmu05010 | Alzheimer disease | 0.0026 |
| Müller Cells | mmu05208 | Chemical carcinogenesis - reactive oxygen species | 0.0026 |
| Müller Cells | mmu04974 | Protein digestion and absorption | 0.0028 |
| Müller Cells | mmu04915 | Estrogen signaling pathway | 0.0028 |
| Müller Cells | mmu04015 | Rap1 signaling pathway | 0.0028 |
| Müller Cells | mmu04971 | Gastric acid secretion | 0.0028 |
| Müller Cells | mmu05165 | Human papillomavirus infection | 0.0028 |
| Müller Cells | mmu05225 | Hepatocellular carcinoma | 0.0028 |
| Müller Cells | mmu04512 | ECM-receptor interaction | 0.0032 |
| Müller Cells | mmu04810 | Regulation of actin cytoskeleton | 0.0036 |
| Müller Cells | mmu04928 | Parathyroid hormone synthesis, secretion and action | 0.0067 |
| Müller Cells | mmu04014 | Ras signaling pathway | 0.0070 |
| Müller Cells | mmu04713 | Circadian entrainment | 0.0077 |
| Müller Cells | mmu04151 | PI3K-Akt signaling pathway | 0.0082 |
| Müller Cells | mmu05020 | Prion disease | 0.0082 |
| Müller Cells | mmu05100 | Bacterial invasion of epithelial cells | 0.0082 |
| Müller Cells | mmu05132 | Salmonella infection | 0.0082 |
| Müller Cells | mmu04721 | Synaptic vesicle cycle | 0.0088 |
| Müller Cells | mmu05163 | Human cytomegalovirus infection | 0.0090 |
| Müller Cells | mmu05222 | Small cell lung cancer | 0.0122 |
| Müller Cells | mmu05224 | Breast cancer | 0.0130 |
| Müller Cells | mmu04520 | Adherens junction | 0.0147 |
| Müller Cells | mmu00480 | Glutathione metabolism | 0.0159 |
| Müller Cells | mmu04540 | Gap junction | 0.0181 |
| Müller Cells | mmu05214 | Glioma | 0.0181 |
| Müller Cells | mmu04550 | Signaling pathways regulating pluripotency of stem cells | 0.0181 |
| Müller Cells | mmu04260 | Cardiac muscle contraction | 0.0181 |
| Müller Cells | mmu04916 | Melanogenesis | 0.0181 |
| Müller Cells | mmu04933 | AGE-RAGE signaling pathway in diabetic complications | 0.0192 |
| Müller Cells | mmu04727 | GABAergic synapse | 0.0204 |
| Müller Cells | mmu05133 | Pertussis | 0.0212 |
| Müller Cells | mmu05016 | Huntington disease | 0.0266 |
| Müller Cells | mmu04919 | Thyroid hormone signaling pathway | 0.0266 |
| Müller Cells | mmu05146 | Amoebiasis | 0.0266 |
| Müller Cells | mmu04137 | Mitophagy - animal | 0.0266 |
| Müller Cells | mmu00190 | Oxidative phosphorylation | 0.0266 |
| Müller Cells | mmu05022 | Pathways of neurodegeneration - multiple diseases | 0.0267 |
| Müller Cells | mmu04350 | TGF-beta signaling pathway | 0.0283 |
| Müller Cells | mmu04261 | Adrenergic signaling in cardiomyocytes | 0.0294 |
| Müller Cells | mmu04145 | Phagosome | 0.0294 |
| Müller Cells | mmu04921 | Oxytocin signaling pathway | 0.0301 |
| Müller Cells | mmu04140 | Autophagy - animal | 0.0325 |
| Müller Cells | mmu05218 | Melanoma | 0.0338 |
| Müller Cells | mmu04724 | Glutamatergic synapse | 0.0340 |
| Müller Cells | mmu04330 | Notch signaling pathway | 0.0340 |
| Müller Cells | mmu04024 | cAMP signaling pathway | 0.0340 |
| Müller Cells | mmu04970 | Salivary secretion | 0.0340 |
| Müller Cells | mmu04918 | Thyroid hormone synthesis | 0.0369 |
| Müller Cells | mmu04966 | Collecting duct acid secretion | 0.0372 |
| Müller Cells | mmu05210 | Colorectal cancer | 0.0379 |
| Müller Cells | mmu04912 | GnRH signaling pathway | 0.0435 |
| Müller Cells | mmu05415 | Diabetic cardiomyopathy | 0.0469 |
| Müller Cells | mmu04728 | Dopaminergic synapse | 0.0484 |
| Pericytes | mmu04926 | Relaxin signaling pathway | 0.0054 |
| Pericytes | mmu04670 | Leukocyte transendothelial migration | 0.0057 |
| Pericytes | mmu04210 | Apoptosis | 0.0057 |
| Pericytes | mmu04371 | Apelin signaling pathway | 0.0057 |
| Pericytes | mmu05012 | Parkinson disease | 0.0180 |
| Pericytes | mmu04510 | Focal adhesion | 0.0180 |
| Pericytes | mmu04137 | Mitophagy - animal | 0.0180 |
| Pericytes | mmu05222 | Small cell lung cancer | 0.0321 |
| Pericytes | mmu04810 | Regulation of actin cytoskeleton | 0.0355 |
| Pericytes | mmu04713 | Circadian entrainment | 0.0355 |
| Pericytes | mmu05167 | Kaposi sarcoma-associated herpesvirus infection | 0.0355 |
| Pericytes | mmu04010 | MAPK signaling pathway | 0.0355 |
| Pericytes | mmu04270 | Vascular smooth muscle contraction | 0.0466 |
| Pericytes | mmu05012 | Parkinson disease | 0.0003 |
| Pericytes | mmu04714 | Thermogenesis | 0.0004 |
| Pericytes | mmu00190 | Oxidative phosphorylation | 0.0008 |
| Pericytes | mmu04066 | HIF-1 signaling pathway | 0.0025 |
| Pericytes | mmu05208 | Chemical carcinogenesis - reactive oxygen species | 0.0029 |
| Pericytes | mmu04720 | Long-term potentiation | 0.0038 |
| Pericytes | mmu04137 | Mitophagy - animal | 0.0041 |
| Pericytes | mmu05415 | Diabetic cardiomyopathy | 0.0065 |
| Pericytes | mmu04919 | Thyroid hormone signaling pathway | 0.0103 |
| Pericytes | mmu04964 | Proximal tubule bicarbonate reclamation | 0.0123 |
| Pericytes | mmu04727 | GABAergic synapse | 0.0123 |
| Pericytes | mmu04022 | cGMP-PKG signaling pathway | 0.0129 |
| Pericytes | mmu05230 | Central carbon metabolism in cancer | 0.0132 |
| Pericytes | mmu04144 | Endocytosis | 0.0185 |
| Pericytes | mmu03013 | Nucleocytoplasmic transport | 0.0185 |
| Pericytes | mmu00270 | Cysteine and methionine metabolism | 0.0208 |
| Pericytes | mmu04120 | Ubiquitin mediated proteolysis | 0.0233 |
| Pericytes | mmu04260 | Cardiac muscle contraction | 0.0233 |
| Pericytes | mmu05132 | Salmonella infection | 0.0233 |
| Pericytes | mmu04724 | Glutamatergic synapse | 0.0276 |
| Pericytes | mmu05017 | Spinocerebellar ataxia | 0.0333 |
| Pericytes | mmu04922 | Glucagon signaling pathway | 0.0333 |
| Pericytes | mmu04710 | Circadian rhythm | 0.0404 |
| Pericytes | mmu04141 | Protein processing in endoplasmic reticulum | 0.0422 |
| Pericytes | mmu03018 | RNA degradation | 0.0422 |
| Pericytes | mmu04114 | Oocyte meiosis | 0.0426 |
| Pericytes | mmu01212 | Fatty acid metabolism | 0.0431 |
| Pericytes | mmu05225 | Hepatocellular carcinoma | 0.0431 |
| Pericytes | mmu05231 | Choline metabolism in cancer | 0.0440 |
| Pericytes | mmu04911 | Insulin secretion | 0.0440 |
| Pericytes | mmu05215 | Prostate cancer | 0.0464 |
| Pericytes | mmu00310 | Lysine degradation | 0.0469 |

**Supplementary Table 2. The KEGG analysis enrichment pathways of the four types of cells inMicroglial Cells**

| Cell type | ID | Description | p.adjust | Count | Expression |
| --- | --- | --- | --- | --- | --- |
| Microglial Cells 1 | mmu04714 | Thermogenesis | <0.00001 | 45 | down |
| Microglial Cells 1 | mmu04810 | Regulation of actin cytoskeleton | 0.0264 | 12 | down |
| Microglial Cells 1 | mmu05208 | Chemical carcinogenesis - reactive oxygen species | <0.00001 | 45 | down |
| Microglial Cells 1 | mmu04722 | Neurotrophin signaling pathway | 0.0261 | 9 | up |
| Microglial Cells 1 | mmu04640 | Hematopoietic cell lineage | 0.0193 | 8 | up |
| Microglial Cells 1 | mmu04933 | AGE-RAGE signaling pathway in diabetic complications | 0.0032 | 10 | up |
| Microglial Cells 1 | mmu05418 | Fluid shear stress and atherosclerosis | 0.0006 | 14 | up |
| Microglial Cells 1 | mmu04151 | PI3K-Akt signaling pathway | 0.0005 | 24 | up |
| Microglial Cells 1 | mmu05417 | Lipid and atherosclerosis | <0.00001 | 20 | up |
| Microglial Cells 1 | mmu04668 | TNF signaling pathway | <0.00001 | 17 | up |
| Microglial Cells 2 | mmu04068 | FoxO signaling pathway | 0.0427 | 6 | down |
| Microglial Cells 2 | mmu04933 | AGE-RAGE signaling pathway in diabetic complications | 0.0146 | 6 | down |
| Microglial Cells 2 | mmu05418 | Fluid shear stress and atherosclerosis | 0.0092 | 8 | down |
| Microglial Cells 2 | mmu04010 | MAPK signaling pathway | 0.0002 | 14 | down |
| Microglial Cells 2 | mmu04621 | NOD-like receptor signaling pathway | 0.0121 | 21 | up |
| Microglial Cells 2 | mmu03050 | Proteasome | <0.00001 | 14 | up |
| Microglial Cells 2 | mmu05208 | Chemical carcinogenesis - reactive oxygen species | <0.00001 | 49 | up |
| Microglial Cells 2 | mmu04714 | Thermogenesis | <0.00001 | 51 | up |
| Microglial Cells 2 | mmu00190 | Oxidative phosphorylation | <0.00001 | 47 | up |
| Microglial Cells 3 | mmu04010 | MAPK signaling pathway | 0.0010 | 18 | up |
| Microglial Cells 3 | mmu05418 | Fluid shear stress and atherosclerosis | 0.0033 | 11 | up |
| Microglial Cells 3 | mmu05417 | Lipid and atherosclerosis | <0.00001 | 17 | up |
| Microglial Cells 3 | mmu03050 | Proteasome | 0.0448 | 4 | down |
| Microglial Cells 3 | mmu04217 | Necroptosis | 0.0225 | 9 | down |
| Microglial Cells 3 | mmu04933 | AGE-RAGE signaling pathway in diabetic complications | 0.0134 | 7 | down |
| Microglial Cells 3 | mmu05208 | Chemical carcinogenesis - reactive oxygen species | 0.0002 | 15 | down |
| Microglial Cells 3 | mmu04210 | Apoptosis | 0.0002 | 12 | down |
| Microglial Cells 3 | mmu04714 | Thermogenesis | 0.0001 | 16 | down |
| Microglial Cells 3 | mmu00190 | Oxidative phosphorylation | <0.00001 | 13 | down |
| Microglial Cells 4 | mmu04933 | AGE-RAGE signaling pathway in diabetic complications | 0.0037 | 18 | down |
| Microglial Cells 4 | mmu04611 | Platelet activation | 0.0007 | 23 | down |
| Microglial Cells 4 | mmu04010 | MAPK signaling pathway | <0.00001 | 52 | down |
| Microglial Cells 4 | mmu05417 | Lipid and atherosclerosis | <0.00001 | 44 | down |
| Microglial Cells 4 | mmu04210 | Apoptosis | <0.00001 | 33 | down |
